# The Impact of Beta Blockers on Survival in Cancer Patients: A Systematic Review and Meta-Analysis

**DOI:** 10.3390/cancers17081357

**Published:** 2025-04-18

**Authors:** Alisha E. Sharma, Stephanie Chan, Adam S. Komorowski, Xingshan Cao, Yizhuo Gao, Kushal Kshatri, Kairavi Desai, Markus Kuksis, Michael Rosen, Anjali Sachdeva, Isabella Kojundzic, Saif Samari, Iacovos P. Michael, Husam Abdel-Qadir, Katarzyna J. Jerzak

**Affiliations:** 1 Department of Medicine, University of Toronto, Toronto, ON M5S 3H2, Canada; alisha.sharma@medportal.ca; 2 Schulich School of Medicine and Dentistry, University of Western Ontario, London, ON N6A 5C1, Canada; 3 Department of Health Research Methods, Evidence, and Impact, McMaster University, Hamilton, ON L8N 3Z5, Canada; 4 Department of Research Design and Biostatistics, Sunnybrook Health Sciences Centre, Toronto, ON M4N 3M5, Canada; 5 Cumming School of Medicine, University of Calgary, Calgary, AB T2N 2T8, Canada; 6 Department of Medicine, McMaster University, Hamilton, ON L8N 3Z5, Canada; 7 Michael G DeGroote School of Medicine, McMaster University, Hamilton, ON L8P 1H6, Canada; 8 Department of Family Medicine, University of Toronto, Toronto, ON M5G 1V7, Canada; 9 Department of Anesthesiology, Perioperative and Pain Medicine, University of Manitoba, Winnipeg, MB R3E 0W2, Canada; 10 Temerty Faculty of Medicine, University of Toronto, Toronto, ON M5S 3H2, Canada; 11 Faculty of Medicine, University of Ottawa, Ottawa, ON K1H 8M5, Canada; 12 Sunnybrook Research Institute, Toronto, ON M4N 3M5, Canada; 13 Institute of Health Policy, Management, and Evaluation, University of Toronto, Toronto, ON M5T 3M6, Canada; 14 Division of Medical Oncology, Sunnybrook Odette Cancer Centre, University of Toronto, Toronto, ON M4N 3M5, Canada

**Keywords:** cardio-oncology, drug repurposing, pharmacology, beta blockers

## Abstract

Background/Objectives: Beta adrenergic signaling has been implicated in cancer progression, leading to interest in repurposing beta blockers (BBs) as adjunctive anti-cancer agents. However, clinical findings are inconsistent. This systematic review and meta-analysis evaluates the association between BB use and survival outcomes in cancer patients. Methods: A systematic search of OVID Medline, EMBASE, and CENTRAL was conducted through 13 September 2023, for studies comparing survival outcomes in solid tumor patients using BBs versus non-users. Eligible studies reported hazard ratios (HRs) for overall survival (OS), progression-free survival (PFS), or cancer-specific survival (CSS). Perioperative studies and those without BB-specific HRs were excluded. Data extraction and quality assessment were performed in duplicate using ROBINS-I. A random-effects model was used, with heterogeneity assessed by the I^2^ statistic. Results: Seventy-nine studies (492,381 patients) met the inclusion criteria; 2.5% were prospective. The most frequently studied cancers were breast (*n* = 33), ovarian (*n* = 30), and colorectal (*n* = 28). BB use was associated with improved PFS (HR 0.78, 95% CI: 0.66–0.92, I^2^ = 79.8%), with significance maintained after excluding high-bias studies (HR 0.74, 95% CI: 0.61–0.91, I^2^ = 36.6%). No significant associations were observed for OS (HR 0.99, 95% CI: 0.94–1.04, I^2^ = 84.9%) or CSS (HR 0.95, 95% CI: 0.91–1.00, I^2^ = 77.4%). Conclusions: BB use may be associated with longer PFS in cancer patients, but findings are limited by study design and heterogeneity; high-quality prospective studies are needed.

## Simple Summary

There is growing interest in whether commonly used medications, such as beta blockers, could have benefits beyond their historical indications for heart conditions. Some studies suggest that beta blockers may slow cancer progression, but research findings have been mixed thus far. This study systematically reviewed and analyzed existing data to better understand whether beta blocker use may be linked to improved survival in cancer patients. We examined data from nearly half a million patients across multiple cancer types, assessing key survival outcomes. Our findings suggest that beta blocker use may be associated with a longer interval between cancer onset and progression. However, no clear benefit was seen for overall survival or cancer-specific survival. Ultimately, more high-quality research is needed. These results contribute to the ongoing discussion about repurposing existing medications for cancer treatment and highlight the need for further investigation through well-designed clinical trials.

## 1. Introduction

**Figure 1:**
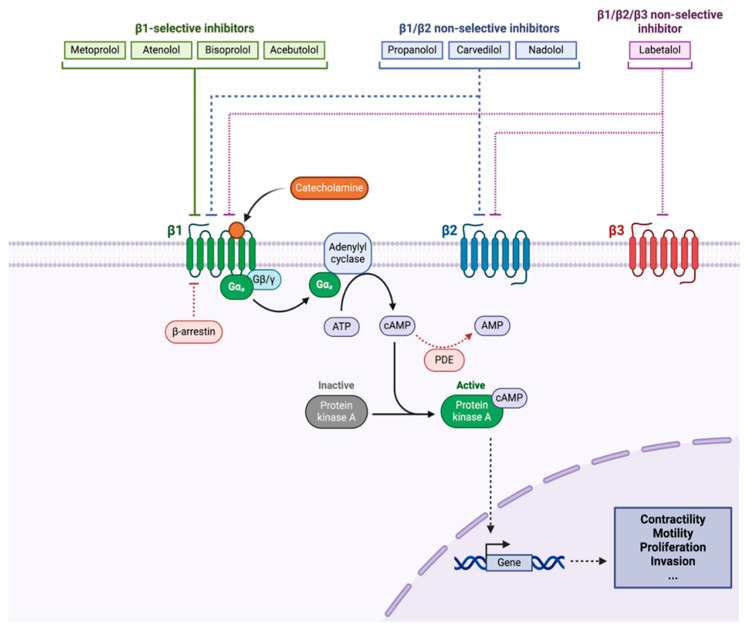
Beta adrenergic receptor signaling pathway. Legend: ATP, adenosine triphosphate; cAMP, cyclic adenosine monophosphate; PDE, phosphodiesterase; PKA, protein kinase A; Gαs, G-alpha subunit; Gβ/γ, G-beta/gamma subunits; β1, β2, β3, beta-adrenergic receptors.

The sympathetic nervous system governs physiological stress response primarily through the release of catecholamines, epinephrine and norepinephrine, from the adrenal medulla. These catecholamines activate adrenergic receptors on target cells throughout the body to exert an immune regulatory effect, which is mediated predominantly by beta-adrenergic receptors [1], and influence the proliferation, invasion, and metastatic abilities of cancer cells. In addition to systemic tumorigenic effects, the beta blockade also suppresses tumor innervation, which plays a crucial role in tumor progression and response to therapies [2]. BBs have long been used for the treatment of cardiovascular and other medical conditions. More recently, the role of beta-adrenergic signaling in multiple processes driving tumor progression [3] has been highlighted by preclinical studies [1,3,4,5,6] (Figure 1), generating growing clinical interest in the potential repurposing of BBs as adjunctive anti-cancer agents. However, observational studies thus far have yielded mixed conclusions regarding their potential clinical use in oncology.

Several mechanisms focusing on beta adrenergic receptor (BAR) signaling have been proposed in an attempt to elucidate the role of BBs in tumor growth (Figure 1). Tumor cell invasion and remodeling of the microenvironment have been directly related to BAR signaling leading to increased invasion and metastasis in animal models of solid tumor [4,5]. Similar effects have also been observed in the progression of leukemia [6] and B-cell lymphoma [3] mouse models. BBs suppress beta-adrenergic signaling, thus potentially mitigating cancer progression through suppression of the downstream proliferation, angiogenesis and metastasis of tumor cells.

While no robust randomized controlled trial evidence exists, multiple retrospective analyses have investigated the association between BB use and clinical outcomes among cancer patients, with varying conclusions. Variations in results may be due to differences in the specific BBs being evaluated, differences in the population being studied (cancer type, stage of disease, extent of cardiac comorbidity resulting in BB use), as well as design/methodological aspects such as immortal time bias (ITB), co-interventions and other confounding variables (e.g., underlying medical conditions necessitating the use of cardiac protective agents).

The main objective of this systematic review and meta-analysis was to provide a comprehensive synthesis of the current evidence on the association between BB exposure and cancer outcomes, specifically considering the effect of ITB.

## 2. Materials and Methods

With the assistance of a research librarian, we systematically searched OVID Medline, EMBASE, and Cochrane Central Register of Controlled Trials for English articles from database inception until 13 September 2023 using MeSH subject headings and keywords, as detailed in Supplement S1. Conference abstracts and reference lists of included and relevant studies identified in this search were also searched for potentially eligible studies. Studies were eligible for inclusion if they presented original research comparing any cancer patients using BBs to patients not using BBs for outcomes of progression-free survival (PFS), cancer-specific survival (CSS), and/or overall survival (OS). Eligible study designs included observational studies, retrospective studies, prospective RCTs, quasi-experimental studies, case–control, and cohort studies. Reviews and meta-analyses, case reports, commentaries, in vitro studies, and abstracts without full-text manuscripts were excluded. Non-English studies, animal studies, pediatric studies, and studies with non-solid tumor cancers were excluded. Studies investigating the risk of developing cancer rather than cancer outcomes, as well as studies investigating cardiac-specific rather than survival outcomes, were excluded.

One reviewer (AES) performed the search. Abstract screening and full text review were performed blinded, using Rayyan [7], and in duplicate, with discrepancies resolved by consensus or adjudication by a third author. SC, MR, MK, AES, and IK performed abstract screening; SC, MR, AES, and IK performed full text screening; MK provided adjudication for full-text screening where required. Data extraction was conducted independently by four authors (SC, MK, AES, IK). Discrepancies were resolved through discussion, or by a fifth author (MR) when consensus could not be reached. Extracted data included study type, study methodology, BB use definition, patient demographics, cancer type and stage, and statistical outcomes (HR, 95% CI, *p*-values). For cancer stage, analyses in which stage was defined as ‘recurrent/metastatic’ or ‘unresectable’ were classified as ‘advanced/metastatic’. The classification of ‘any stage’ was applied for studies which included all cancer stages, as well as those which did not clearly specify cancer stage.

Data for all survival analyses reporting a corresponding hazard ratio (HR) with 95% confidence interval (CI) for a distinct cancer type were extracted and covariates were recorded. BB use was characterized by time of use relative to cancer diagnosis. In studies including multiple different definitions of BB use, studies analyzing BB use as a time varying covariate were used preferentially. Otherwise, analyses of pre-diagnostic BB use were favored over post-diagnostic use to limit the risk of ITB. Additionally, when multiple data sets were presented for the same population and outcome, the model adjusting for the most confounders was included in the meta-analysis. Further, data sets accounting for a larger patient population were favored over analyses with smaller patient populations within a single study. Studies specifically investigating incidental BB use within the perioperative period were excluded from this meta-analysis to reduce heterogeneity and avoid potential confounding associated with the perioperative stress response. Outcomes other than cancer-specific survival, PFS/recurrence-free survival (RFS), and all-cause mortality (ACM)/OS were uncommon among included studies and therefore were excluded from the meta-analysis. Studies without BB-specific HRs were excluded. If multiple HRs for distinct outcomes and/or cancer patient populations were presented in a single study, all relevant and eligible analyses were included.

Potential patient population overlap between eligible studies was identified based on shared data source, primary cancer, and study period. In cases of population overlap between studies of similar methodology, the analysis with the most complete data set was included for each outcome. In the case of population overlap between identical analyses, the most recent study was included.

HRs and 95% CIs were calculated using a random-effects model with inverse variance weighting using the DerSimonian and Laird method [8] to pool estimates; the Jackson method was used to calculate the confidence interval for the *τ*^2^ statistic. RStudio (Version 2023.06.1. Built 524) [9] was used for analyses. Heterogeneity was assessed using the I^2^ statistic and the Q-statistic. Individual and pooled effect sizes and confidence intervals were presented using forest plots. Primary analyses compared OS, CSS, and PFS in users of any BBs to that of non-users. Additional analyses explored these outcomes in users of selective BBs vs. non-users, and non-selective BB users vs. non-users. The influence of cancer type and cancer stage was studied in two separate exploratory subgroup analyses. A sensitivity analysis was performed in which the primary analyses were repeated excluding studies with risk of ITB, in order to assess the influence of ITB on survival outcomes.

Risk of bias was assessed blinded and in duplicate (SS, KD, KK, ALS) using the Risk Of Bias In Non-Randomized Studies of Interventions (ROBINS-I) tool. Discrepancies were resolved initially by discussion, or through adjudication by a third reviewer (ASK) when consensus could not be reached. Descriptive statistics were used to summarize the risk of bias within and across studies. The Chi-square test was used with a significance level of 0.05. The certainty of evidence was assessed using the GRADE approach. A summary of findings table was generated using the GRADEpro app [10] and presented using standardized terms [11].

BB use was categorized as either pre-diagnosis, post-diagnosis, or at diagnosis, when this information was available, to establish the risk of immortal time bias in each study. For studies that did not provide this information, the risk of immortal bias was defined as ‘unclear’. We assessed for publication bias through the use of funnel plots for analyses containing more than 10 studies, using visual inspection to assess for asymmetry.

The protocol for this systematic review and meta-analysis was prospectively registered with PROSPERO (CRD42020200238) and reported according to PRISMA 2020 guidelines [12]. There were several notable protocol amendments after registration: the search end date was amended from 29 May 2020 to 13 September 2023, and the ROBINS-I tool [13] was used in place of the Newcastle–Ottawa Scale.

There was no funding source for this study.

## 3. Results

**Figure 2:**
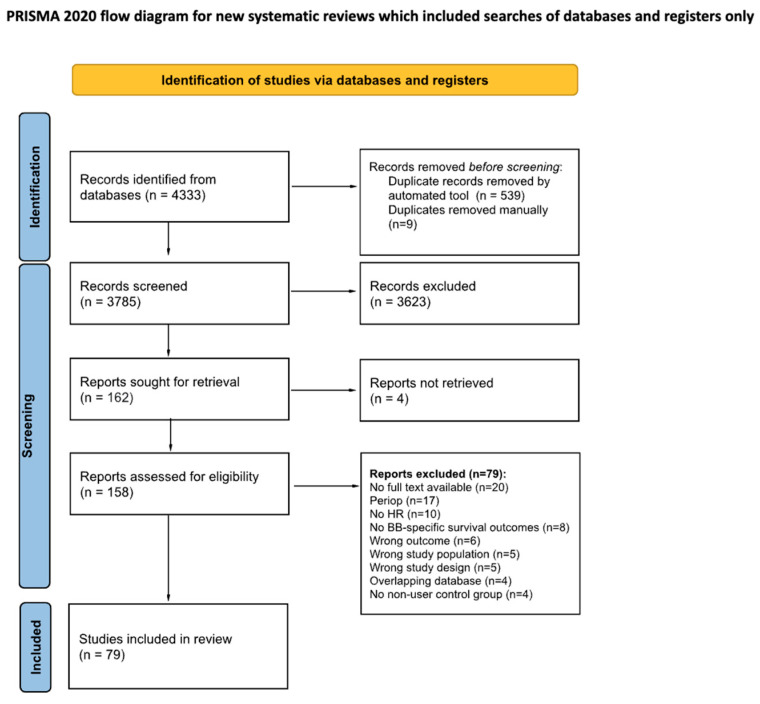
PRISMA diagram [12].

A total of 4333 articles were identified through the search of databases up to 13 September 2023. After deduplication, 3785 articles remained. In total, 3623 articles were excluded after title and abstract screening, and an additional 79 articles were excluded after full-text screening. A total of 79 articles [14,15,16,17,18,19,20,21,22,23,24,25,26,27,28,29,30,31,32,33,34,35,36,37,38,39,40,41,42,43,44,45,46,47,48,49,50,51,52,53,54,55,56,57,58,59,60,61,62,63,64,65,66,67,68,69,70,71,72,73,74,75,76,77,78,79,80,81,82,83,84,85,86,87,88,89,90,91,92] were ultimately included in the systematic review and meta-analysis (Figure 2).

### 3.1. Study Characteristics

We identified 79 eligible studies comprising 213 distinct analyses and 492,381 total patients (Supplemental Table S1). All included studies were observational; 2/79 were prospective (2.5%) and the remainder were retrospective. The most common primary tumor types assessed included breast (n = 33), ovarian (n = 30), and colorectal (n = 28) cancers. Among analyses which specified disease stage, advanced disease (n = 56) was more commonly studied than early/non-metastatic disease (n = 26).

### 3.2. Progression-Free Survival

**Figure 3:**
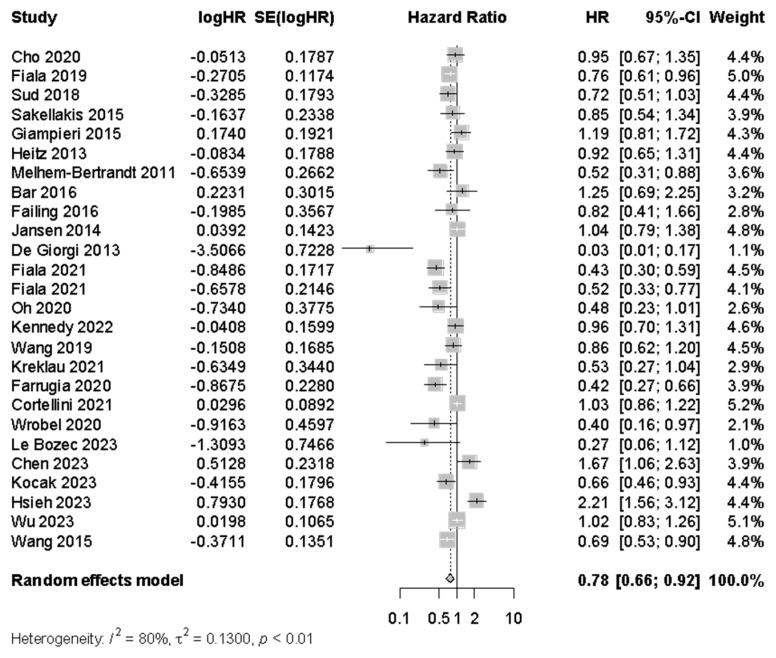
Progression-free survival among cancer patients who were beta blocker users versus non-users.

In total, 26 analyses compared PFS with any and no BB use (Figure 3), with a statistically significant difference identified in PFS between the two groups (HR 0.78 [95% CI: 0.66–0.92]). I^2^ of 79.8% suggested a substantial level of heterogeneity in this analysis. Egger’s test indicated significant funnel plot asymmetry (*p* = 0.03) with a bias coefficient of −2.26, suggesting potential publication bias.

**Figure 4:**
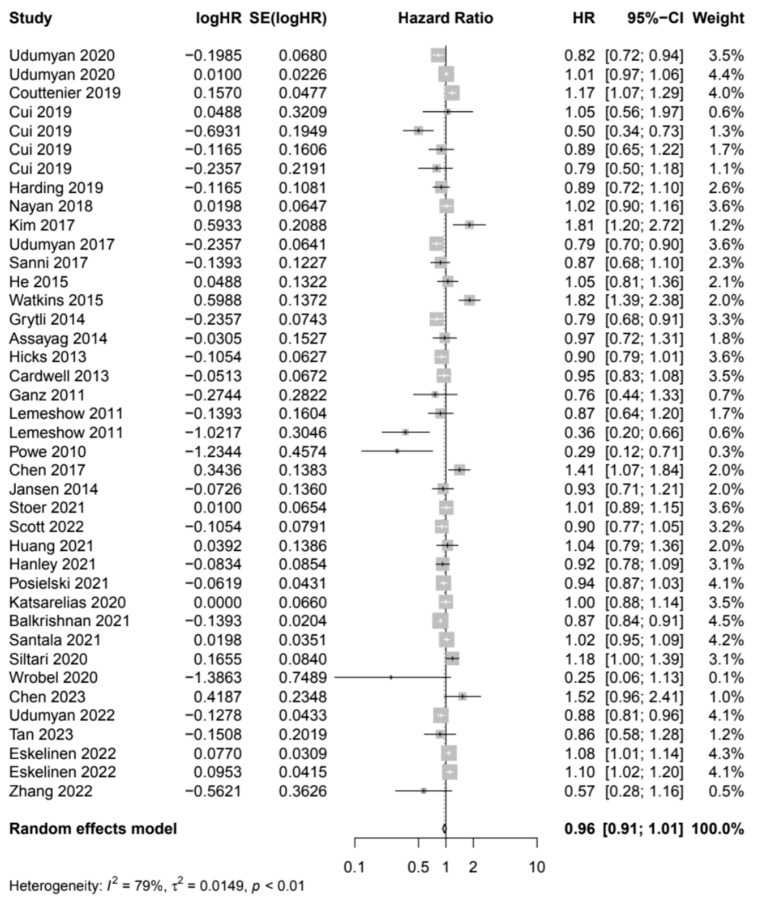
Cancer-specific survival among cancer patients who were beta blocker users versus non-users.

Results for analyses of any vs. no BB use in relation to CSS were reported in 39 studies (Figure 4) and meta-analysis revealed a trend for longer CSS, although this was not statistically significant (HR 0.95: CI 95%: 0.91–1.00). There was substantial heterogeneity in this analysis, with I^2^ of 77.4%. Egger’s test showed no significant publication bias (*p* = 0.61).

**Figure 5:**
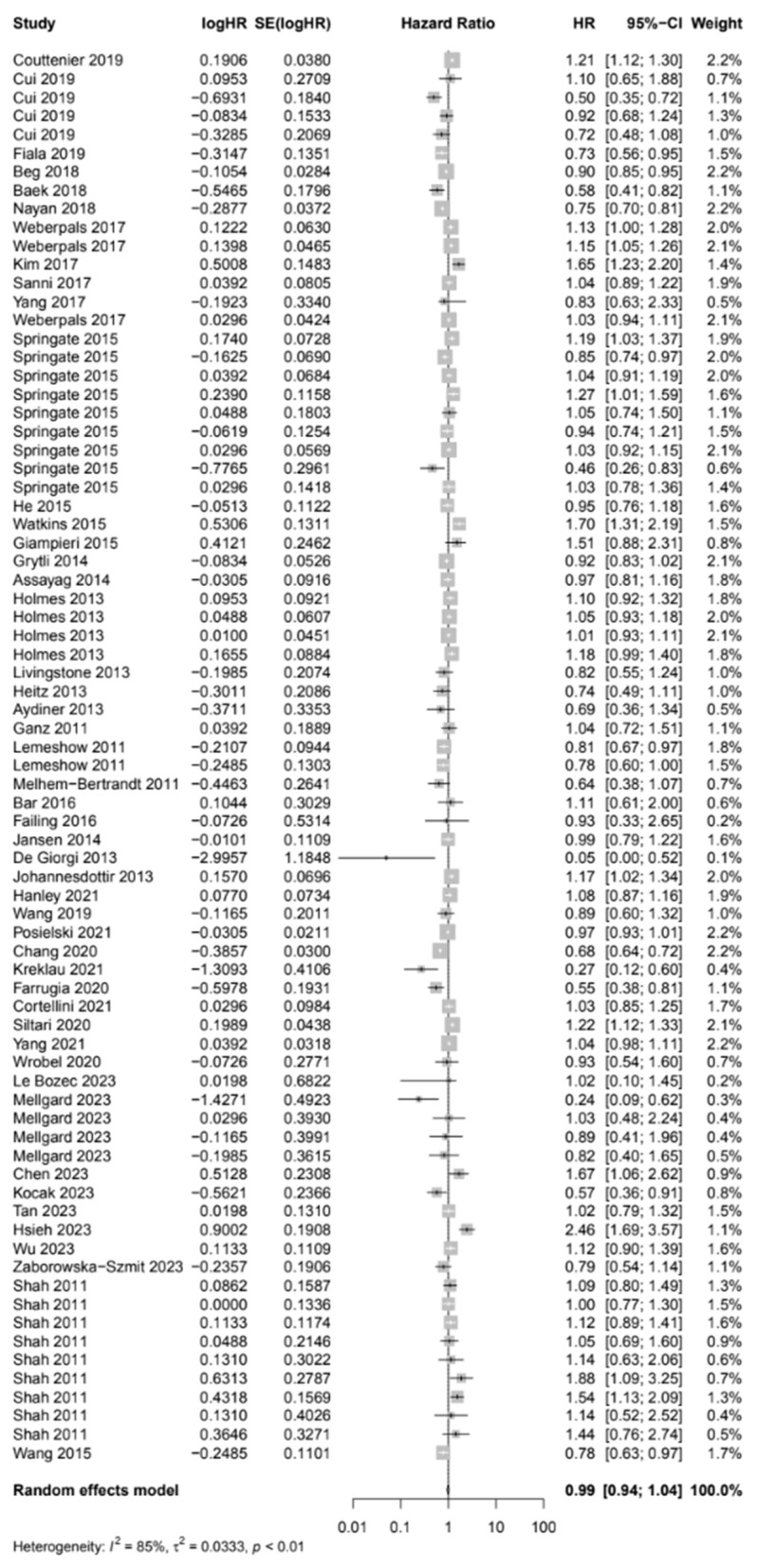
Overall survival among cancer patients who were beta blocker users versus non-users.

Results from 76 reports provided analysis pertaining to OS in users of any type of BB to non-users (Figure 5), with a pooled HR of 0.99 (95% CI: 0.94–1.04). Heterogeneity analysis revealed substantial variability between studies (I^2^ = 84.9%). Egger’s test showed no evidence of publication bias (*p*-value = 0.71).

**Figure 6:**
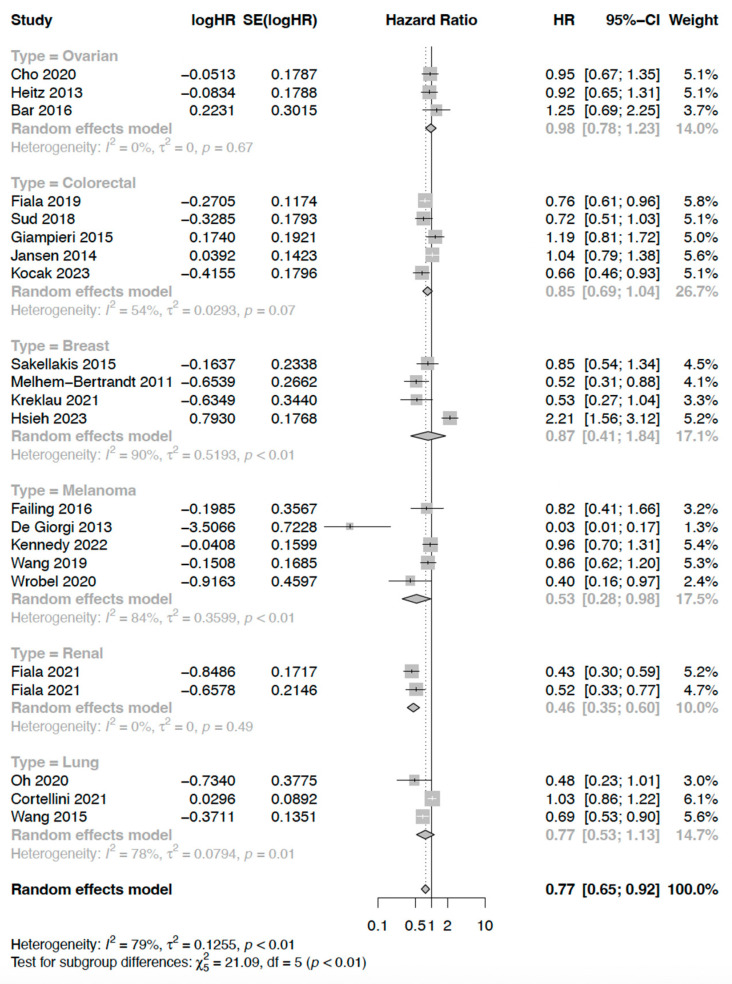
Progression-free survival subgroup by cancer type.

Subgroup analysis of PFS found that the association between BB use and longer PFS was significant across patients with early-stage (n = 2, HR 0.62 [95% CI: 0.40–0.96]) and advanced (n = 15, HR 0.87 [0.80–0.94]) cancers, although conclusions regarding the former are limited by small subgroup size. In subgroup analysis by cancer type (Figure 6), the beta blockade was associated with longer PFS in patients with melanoma (n = 5, HR 0.53 [95% CI: 0.28–0.98]), but was not significantly associated with PFS in other cancers, including colorectal (n = 5, HR = 0.85 [95% CI: 0.69–1.04]) and breast (n = 4, HR = 0.87 [95% CI: 0.41–1.84]). Subgroup analyses by BB type were limited because only eight studies specified the use of either selective (n = 3) or non-selective (n = 5) BBs.

An exploratory analysis was performed to determine whether the association between BB and CSS differed in populations of patients with specific malignancies. The beta blockade was associated with longer CSS in patients with colorectal cancer (n = 5, HR 0.83 [95% CI: 0.73–0.95]) and shorter CSS in patients with head and neck (n = 2, HR 1.68 [95% CI: 1.23–2.28]) and renal cancer (n = 3, HR 1.08 [95% CI: 1.03–1.13]). In subgroup analysis by BB type, neither selective beta blockade (n = 14, HR 0.95 [95% CI: 0.89–1.01) nor non-selective beta blockade (n = 14, HR 0.85 [95% CI: 0.72–1.01]) were significantly associated with CSS.

Of the 79 reports reviewed, risk of bias assessments using the ROBINS-I tool indicated that 38 (48.1%) studies were at moderate risk of overall bias, 36 reports (45.6%) were at serious risk of overall bias, and 5 (6.3%) were at critical risk of overall bias. Detailed assessment of potential sources of biases using the ROBINS-I tool for each outcome is provided in Supplemental Table S2. There was no significant difference in these domains across outcomes. With regard to ITB, 22/34 (64.7%) of the included analyses for PFS, 13/70 (18.6%) for CSS, and 76/109 (69.7%) for OS were felt to be at risk.

Overall, the certainty of evidence was graded as very low for PFS, which was downgraded for the presence of publication bias, and low for CSS and OS, due largely to their observational nature.

**Figure 7:**
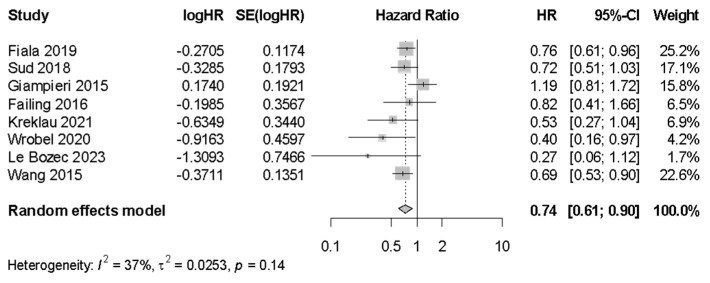
Progression-free survival sensitivity analysis for immortal time bias.

Sensitivity analysis was performed after exclusion of studies identified as being at risk of ITB. In this analysis (Figure 7), there remained a significant association between BB use and longer PFS (n = 8, HR 0.74 [95% CI 0.61–0.90], I^2^ = 36.6%). Similar sensitivity analyses for CSS (n = 33, HR 1.01 [95% CI: 0.88–1.02]) and OS (n = 54, HR 1.00 [95% CI: 0.93–1.06], I^2^ = 86.8%) when excluding studies at risk of ITB did not demonstrate significant associations.

## 4. Discussion

Overall, our review suggests that BB use may be associated with a longer PFS among patients with cancer, irrespective of cancer type or stage; these findings remained statistically significant after excluding studies at risk of ITB. In the overall cohort, there was a trend for association between BB use and longer CSS, but no difference in OS.

While PFS should not be universally considered a surrogate for OS, it remains a clinically meaningful outcome which is increasingly used both as a regulatory endpoint for the approval of cancer therapies and as a primary endpoint in oncology RCTs [93]. The observed PFS benefit suggests that beta blockade may have the potential to delay cancer progression, which is of particular interest given the already widespread use of beta blockers.

Given that BBs are most commonly prescribed to patients with cardiovascular disease, BB users are likely at higher risk of death than non-users; hence, the lack of OS detriment in the included observational studies is notable. Indeed, patients with a primary indication for BBs may be predisposed to worse outcomes due to the inherently higher risk of competing events, including myocardial infarction, death from vascular causes, and stroke, often associated with conditions for which beta blockade is indicated. Conversely, these competing risks may provide a theoretical protective effect for PFS and CSS, as patients with competing risks resulting in death secondary to CVD would preclude cancer-related death or disease progression [94]. This is less likely to explain the observed PFS benefit in this meta-analysis, but in the absence of a similarly prolonged CSS. The infrequent reporting of CSS (n = 39, 18.3%) and challenging nature of establishing the specific cause of death in this patient population, particularly within the subset of patients with advanced and metastatic disease, also complicates the interpretation of these findings. These findings do not definitively rule out a BB-associated OS or CSS benefit, but rather highlight the need for randomized studies which can appropriately mitigate the influence of confounding variables and thus clarify the impact of long-term BB use.

Past meta-analyses have yielded discrepant results on the effect of beta blockade on PFS [95,96], CSS [95,97,98,99], and OS [100,101,102]. This meta-analysis builds on these studies by examining a broader and more recent body of literature than prior meta-analyses. Discrepancies between prior reviews may also be due in part to the considerable heterogeneity between observational studies, which form the evidence base in this field, given that there are no published randomized clinical trials on this topic. The strengths of this study include the large sample size and consideration of the influence of ITB.

However, several limitations should be considered. These include the observational and predominantly retrospective nature of the included studies. Observational studies are inherently more predisposed to bias than RCTs, as reflected by the at least moderate risk of bias present in each study according to ROBINS-I assessment (Supplemental Table S2). Confounding is also a concern, particularly given that patients prescribed BBs are likely to have cardiovascular comorbidities which may influence both cancer-associated and overall prognosis. Although many studies attempted to control for confounding, statistical methods used for this purpose varied significantly. We aimed to minimize confounding by prioritizing the inclusion of the models that adjusted for the highest number of variables including those related to patient comorbidities, demographics, cancer type, and stage when multiple HRs were presented. However, residual confounding remains a concern due to the variability in methods of adjustment across studies and the potential that comorbidity data collected in retrospective analyses may not have been fully characterized.

Variability across the retrospective studies, which represent the great majority (n = 74/76) studies in this meta-analysis, has been attributed in part to the potential influence of ITB. ITB is thought to inflate the potential survival benefits noted in observational data by classifying patients as users only after they have survived long enough to receive the medication of interest [103]. To mitigate the potential influence of ITB, analyses studying BB use as a time varying covariate were used preferentially over analyses of pre-diagnostic BB use, which were favored over post-diagnostic use.

We also conducted sensitivity analyses excluding studies at high risk of ITB, which demonstrated that the observed PFS benefit conferred by any BB use did not change significantly after exclusion of studies at risk of ITB. This suggests that heterogeneity of PFS is not solely attributable to ITB; heterogeneity may instead have been due in part to variations in study design, definition of PFS, sample sizes, cancer types, beta blocker formulations, and follow-up durations. Sensitivity analysis of CSS and OS demonstrated widened CIs, suggesting that ITB may have marginally inflated the initial results, although this did not reach statistical significance. This highlights the need for critical evaluation of observational study designs, and increasing awareness of the role of ITB in these studies. Notably, the PFS analysis was limited by the presence of publication bias resulting in very low certainty of evidence according to the GRADE system [11]. In addition to publication bias, selection bias may also have led to overestimation of the observed PFS benefit through the exclusion of patients with more aggressive disease with a poor prognosis related to metastatic malignancy. In light of these biases, the observed PFS benefit should be interpreted with caution. CSS and OS were graded as low certainty of evidence, which is the expected level of certainty associated with sound observational studies [104].

We also performed subgroup analyses by BB selectivity, cancer type, and cancer stage to explore potential variations in treatment effects. However, lack of distinction between specific disease stages, cancer types, and BB selectivity in many studies limited the utility of these subgroup analyses. This study also cannot account for the effect of BB use in patients with specific cancer subtypes, as this information was rarely reported in eligible studies. It has also been proposed that BBs may improve survival among cancer patients by potentiating the effects of established therapies including immunotherapy [63,65,105] and chemotherapy [2]. However, this meta-analysis did not specifically investigate this association given inconsistent reporting of concomitant anti-cancer therapy, and available literature remains inconclusive. Similarly, preclinical studies suggest that beta blockade may enhance the effects of radiotherapy [106] in certain patient populations, although supporting evidence is limited. Further preclinical study is needed to clarify the influence of beta blockade in conjunction with different modalities of anti-cancer therapy, as effects may vary depending on the nature of cancer treatment.

Additionally, measuring the impact of BB use across cancer types presents challenges related to variation in cancer biology, growth rate, metastatic potential, and sensitivity to beta-adrenergic signaling. To account for this, subgroup analyses were conducted by cancer type, although interpretation of results was limited by small subgroup sizes. Of note, breast, ovarian, and colorectal cancers were most frequently studied in this meta-analysis. The high representation of breast and colorectal cancers likely reflects their high relative global incidence and associated mortality rates [2,4,5,95]. However, the number of ovarian cancer studies in this meta-analysis appears disproportionate to its relative incidence, which may be multifactorial. First, ovarian cancer often lacks the number of targeted therapy options available for other malignancies [107], increasing interest in identifying alternative therapy options, such as beta blockade, through preclinical and observational studies. Selection bias may also have contributed to this overrepresentation, although this was not explicitly studied in our analysis.

Given the variation in tumor-specific survival outcomes associated with BB use, further study on potential tumor-specific variations in response to beta blockade is warranted. Notably, the median expression of the targets of BBs (ADRB1 and ADRB2 genes encoding beta adrenergic receptors), varies significantly between tumor sites [108]. Differences in response to beta blockade between cancer types may therefore be attributable in part to variable expression of target receptors, with greater BB-associated survival benefit observed in tumors with higher expression of beta adrenergic receptors. Interestingly, although prostate cancer has the highest ADRB1 and ADRB2 expression of the malignancies studied in this meta-analysis [108], beta blockade in prostate cancer was not associated with CSS or OS improvement in this analysis. However, interpretation of this result is difficult due to low subgroup sizes, and future study of BB use in cancer patients may benefit from consideration of beta adrenergic receptor burden in cancer types of interest. A breadth of preclinical evidence highlighting the influence of chronic stress-related beta 2-adrenergic signaling on multiple tumorigenic pathways [109] suggests that ADRB2 expression may be of particular interest, as findings of clinical studies have been variable [63,65,105] thus far.

Currently, ongoing phase II trials (Supplemental Table S3) are investigating the possible adjunctive role of propranolol in the treatment of a number of cancer populations including patients with bladder (NCT04493489), gastric (NCT04005365), and esophageal cancer (NCT04682158), as well as metastatic adenocarcinoma of the esophagus or gastroesophageal junction (NCT05651594). Additionally, a recently registered phase II trial (NCT05741164) seeks to determine the efficacy of propranolol in combination with pembrolizumab in patients with checkpoint inhibitor refractory metastatic TNBC. If favorable, the findings of these trials will lay the foundation for future larger RCTs to establish the highest level of evidence in this field.

## 5. Conclusions

Overall, this comprehensive meta-analysis found that beta blockade may be associated with longer PFS among patients with cancer, which was sustained after the exclusion of studies susceptible to ITB. There was a trend for BB use and longer CSS, but no difference observed for OS in the overall cohort; exploratory analyses by cancer type and stage were limited by small subgroup sizes. BBs currently represent a promising new avenue of adjunctive cancer therapy. In addition to their potential anti-cancer properties, BBs may also provide cardioprotective effects when used in patients receiving cardiotoxic systemic therapies [110]. High-quality evidence from large prospective and randomized studies, including ongoing phase II clinical trials, will ultimately provide a more nuanced understanding of the association between beta blockade and outcomes in specific cancer patient populations and clinical contexts. If positive, these will confirm the utility of BBs as an accessible and cost-effective adjunct to existing cancer therapies.

## Data Availability

The original contributions presented in this study are included in the article/Supplementary Material. Further inquiries can be directed to the corresponding author.

## Supplementary Materials

The following supporting information can be downloaded at: https://www.mdpi.com/article/10.3390/cancers17081357/s1, Table S1: Study Characteristics; S1: Search Strategy; Table S2: ROBINS-I Results; Table S3: Actively recruiting and not yet recruiting trials in the ClinicalTrials.gov database; Table S4: ROBINS-I Summary; Table S5: PRISMA 2020 Checklist [12]; Table S6: Pooled HRs; Figure S1: Subgroup Analysis of PFS by Cancer Stage; Figure S2: Subgroup Analysis of PFS by Non-selective vs. Any Beta Blockers; Figure S3: Subgroup Analysis of PFS by Selective vs. Any Beta Blockers; Figure S4: PFS ITB Sensitivity Analysis; Figure S5: Subgroup Analysis of OS by Cancer Type; Figure S6: Subgroup Analysis of OS by Cancer Stage; Figure S7: Subgroup Analysis of OS by Non-selective vs. Any Beta Blocker Use; Figure S8: Subgroup Analysis of OS by Selective vs. Any Beta Blocker Use; Figure S9: OS ITB Sensitivity Analysis; Figure S10: Subgroup Analysis of CSS by Cancer Type; Figure S12: Subgroup Analysis of CSS by Non-selective vs. Any Beta Blockers; Figure S13: Subgroup Analysis of CSS by Selective vs. Any Beta Blockers; Figure S14: CSS ITB Sensitivity Analysis; Figure S15: OS Publication Bias; Supplemental Figure S16: CSS Publication Bias; Supplemental Figure S17: PFS Publication Bias; Table S7: GRADE Summary of Findings Table [10].
