# Supplementary material for: The Impact of Beta Blockers on Survival in Cancer Patients: A Systematic Review and Meta-Analysis
Source: Cancers (Basel). 2025 Apr 18;17(8):1357. doi: 10.3390/cancers17081357 (PMC12026060; doi:10.3390/cancers17081357)
Supplement: Supplementary file 1 [file cancers-17-01357-s001.zip › cancers-3507749-supplementary.pdf]

Table S1. Study Characteristics ... 2
S1. Search Strategy ... 9
Table S2. ROBINS-I Results ... 14
Table S3. Actively recruiting and not yet recruiting trials in the ClinicalTrials.gov database ... 17
Table S4. ROBINS-I Summary ... 18
Table S5. PRISMA 2020 Checklist ... 19
Table S6. Pooled HRs ... 23
Figure S1. Subgroup Analysis of PFS by Cancer Stage ... 25
Figure S2. Subgroup Analysis of PFS by Non-selective vs Any Beta Blockers ... 25
Figure S3. Subgroup Analysis of PFS by Selective vs Any Beta Blockers ... 25
Figure S4. PFS ITB Sensitivity Analysis ... 26
Figure S5. Subgroup Analysis of OS by Cancer Type ... 27
Figure S6. Subgroup Analysis of OS by Cancer Stage ... 28
Figure S7. Subgroup Analysis of OS by Non-selective vs Any Beta Blocker Use ... 29
Figure S8. Subgroup Analysis of OS by Selective vs Any Beta Blocker Use ... 29
Figure S9. OS ITB Sensitivity Analysis ... 30
Figure S10. Subgroup Analysis of CSS by Cancer Type ... 31
Figure S11. Subgroup Analysis of CSS by Cancer Stage ... 32
Figure S12. Subgroup Analysis of CSS by Non-selective vs Any Beta Blockers ... 32
Figure S13. Subgroup Analysis of CSS by Selective vs Any Beta Blockers ... 32
Figure S14. CSS ITB Sensitivity Analysis ... 33
Figure S15. OS Publication Bias ... 33
Supplemental Figure S16. CSS Publication Bias ... 34
Supplemental Figure S17. PFS Publication Bias ... 34
Table S7. GRADE Summary of Findings Table ... 35

Table S1. Study Characteristics

First Author, Year (n)	Cancer Type	Cancer Stage	Risk of ITB	Beta blocker type	Outcome	HR	95% CI (LL)	95% CI (UL)	P-value
Altshuler, 2022 (n=1444)	Hepatocellular	Advanced/Metastatic	N	Selective	OS	0.75	0.61	0.94	0.01
			N	Selective	PFS	0.66	0.45	0.96	0.03
			N	Non-selective	OS	1.05	0.94	1.32	0.68
			N	Non-selective	PFS	1.17	0.76	1.78	0.48
Assayag, 2014 (n=6270)	Prostate	Early/Non-metastatic	N	Any	CSS	0.97	0.72	1.31	
			N	Non-selective	CSS	1.05	0.72	1.53	
			N	Any	OS	0.97	0.81	1.16	
			N	Non-selective	OS	0.94	0.74	1.18	
Aydiner, 2013 (n=107)	Lung	Advanced/Metastatic	Y	Any	OS	0.69	0.36	1.34	0.27
Baek, 2018 (n=866)	Ovarian	Any stage	Y	Any	OS	0.579	0.407	0.823	0.002
			Y	Selective	OS	0.523	0.332	0.827	0.005
			Y	Non-selective	OS	0.565	0.377	0.848	0.006
Balkrishnan, 2021 (n=13982)	Colorectal	Early/Non-metastatic	N	Any	CSS	0.87	0.84	0.91	
Bar, 2016 (n=143)	Ovarian	Any stage	Y	Any	OS	1.11	0.61	2.00	0.744
			Y	Any	PFS	1.25	0.69	2.25	0.457
Barron, 2011 (n=5801)	Breast	Any stage	N	Non-selective	CSS	0.19	0.06	0.6	
			N	Selective	CSS	1.08	0.84	1.4	
Beg, 2018 (n=13702)	Pancreatic	Any stage	Y	Any	OS	0.90	0.85	0.95	
Cardwell, 2013 (n=9817)	Breast	Any stage	N	Any	CSS	0.95	0.83	1.08	
Cardwell, 2016 (n=133, 251)	Breast	Any stage	N	Non-selective	CSS	1.01	0.85	1.20	0.90
			N	Non-selective	OS	1.16	1.02	1.32	0.02
Chang, 2019 (n=4680)	Hepatocellular	Advanced/Metastatic	N	Non-selective	OS	0.78	0.72	0.84	<0.001
Chang, 2020 (n=4988)	Lung	Advanced/Metastatic	N	Any	OS	0.68	0.64	0.72	<0.001
Chen, 2017 (n=14766)	Breast	Early/Non-metastatic	N	Any	CSS	1.41	1.07	1.84	

Chen, 2023 (n=4192)	Head & Neck	Any stage	Y	Any	OS	1.67	1.06	2.62	0.027
			Y	Any	CSS	1.52	0.96	2.41	0.072
			Y	Any	PFS	1.67	1.06	2.63	0.027
Cho, 2020 (n=878)	Ovarian	Any stage	Y	Any	PFS	0.95	0.67	1.35	0.770
Cortellini, 2021 (n=950)	Lung	Advanced/Metastatic	Y	Any	OS	1.03	0.85	1.25	0.7085
			Y	Any	PFS	1.03	0.86	1.22	0.726
Couttenier, 2019 (n=6197)	Ovarian	Any stage	N	Any	CSS	1.17	1.07	1.29	<0.001
			N	Selective	CSS	1.14	1.03	1.25	0.01
			N	Non-selective	CSS	1.19	1.04	1.36	0.01
			N	Any	OS	1.21	1.12	1.30	<0.001
			N	Selective	OS	1.17	1.08	1.27	<0.001
			N	Non-selective	OS	1.2	1.07	1.34	0.00
Cui, 2019 (n-2891)	Breast	Any stage	N	Any	CSS	1.05	0.56	1.97	
	Colorectal	Any stage	N	Any	CSS	0.50	0.34	0.73	
	Lung	Any stage	N	Any	CSS	0.89	0.65	1.22	
	Upper GI	Any stage	N	Any	CSS	0.79	0.50	1.18	
	Breast	Any stage	N	Any	OS	1.10	0.65	1.88	
	Colorectal	Any stage	N	Any	OS	0.50	0.35	0.72	
	Lung	Any stage	N	Any	OS	0.92	0.68	1.24	
	Upper GI	Any stage	N	Any	OS	0.72	0.48	1.08	
De Giorgi, 2013 (n=741)	Melanoma	Early/Non-metastatic	N	Any	OS	0.05	0.005	0.52	0.01
			Y	Any	PFS	0.03	0.01	0.17	<0.001
De Giorgi, 2018 (n=53)	Melanoma	Early/non-metastatic	N	Non-selective	PFS	0.18	0.04	0.89	0.03
			N	Non-selective	OS	0.64	0.1	3.96	0.63
Eskelinen, 2022 (n=13873)	Renal	Any stage	N	Any	CSS	1.08	1.01	1.14	
		Advanced/Metastatic	N	Any	CSS	1.1	1.02	1.2	
Failing, 2016 (n=159)	Melanoma	Advanced/Metastatic	N	Any	OS	0.93	0.33	2.65	0.9
			N	Any	PFS	0.82	0.41	1.66	0.59
Farrugia, 2020 (n=291)	Upper GI	Any stage	Y	Any	OS	0.55	0.38	0.81	0.002
			Y	Any	PFS	0.42	0.27	0.66	<0.001
Fiala, 2019 (n=514)	Colorectal	Advanced/Metastatic	N	Any	OS	0.730	0.560	0.951	0.020
			N	Any	PFS	0.763	0.606	0.960	0.021
Fiala, 2021 (n-343)	Renal	Advanced/Metastatic	Y	Any	PFS	0.428	0.302	0.592	0.001
			Y	Any	PFS	0.518	0.332	0.77	0.001
Ganz, 2011 (n=1779)	Breast	Early/Non-metastatic	Y	Any	CSS	0.76	0.44	1.33	0.34
			Y	Any	OS	1.04	0.72	1.51	0.83

Giampieri, 2015 (n=235)	Colorectal	Advanced/Metastatic	N	Any	OS	1.51	0.88	2.31	0.14
			N	Any	PFS	1.19	0.81	1.72	0.38
Gillis, 2021 (n=4014)	Breast	Early/Non-metastatic	N	Selective	CSS	0.41	0.15	1.09	0.076
Grytli, 2014 (n=3561)	Prostate	Advanced/Metastatic	N	Any	CSS	0.79	0.68	0.91	0.001
			N	Selective	CSS	0.82	0.70	0.95	0.008
			N	Any	OS	0.92	0.83	1.02	0.11
Hanley, 2021 (n-4207)	Ovarian	Any stage	N	Any	CSS	0.92	0.78	1.09	
			N	Any	OS	1.08	0.87	1.16	
Harding, 2019 (n=1414)	Ovarian	Any stage	N	Any	CSS	0.89	0.72	1.10	
			N	Non-selective	CSS	0.60	0.43	0.83	
			N	Selective	CSS	1.03	0.82	1.30	
He, 2015 (n=1174)	Upper GI	Any stage	N	Any	CSS	1.05	0.81	1.36	0.73
			N	Any	OS	0.95	0.76	1.18	0.65
Heitz, 2013 (n=381)	Ovarian	Any stage	Y	Any	OS	0.74	0.49	1.11	0.15
			Y	Any	PFS	0.92	0.65	1.31	0.65
Hicks, 2013 (n=4794)	Colorectal	Any stage	N	Any	CSS	0.90	0.79	1.01	0.08
			N	Non-selective	CSS	0.97	0.7	1.35	0.87
Holmes, 2013 (n=15582)	Breast	Any stage	N	Any	OS	1.10	0.92	1.32	0.305
	Colorectal	Any stage	N	Any	OS	1.05	0.93	1.18	0.445
	Lung	Any stage	N	Any	OS	1.01	0.93	1.11	0.752
	Prostate	Any stage	N	Any	OS	1.18	0.99	1.40	0.059
Hsieh, 2023 (n=221)	Breast	Advanced/Metastatic	Y	Any	PFS	2.21	1.56	3.12	<0.001
			Y	Any	OS	2.46	1.69	3.57	<0.001
			Y	Non-selective	PFS	1.88	1.26	2.82	0.195
			Y	Selective	PFS	2.72	1.8	4.12	<0.001
			Y	Non-selective	OS	2.1	1.36	3.25	<0.001
			Y	Selective	OS	2.97	1.91	4.62	<0.001
Huang, 2021 (n=950)	Ovarian	Any stage	N	Any	CSS	1.04	0.79	1.36	
Jansen, 2014 (n=1975)	Colorectal	Any stage	Y	Any	CSS	0.93	0.71	1.21	0.5822
			Y	Selective	CSS	0.85	0.65	1.12	0.2524
			Y	Non-selective	CSS	1.31	0.82	2.08	0.2602
			Y	Any	OS	0.99	0.79	1.22	0.8981
			Y	Selective	OS	0.88	0.70	1.10	0.2529

			Y	Non-selective	OS	1.42	0.98	2.05	0.0648
			Y	Any	PFS	1.04	0.79	1.38	0.7604
			Y	Selective	PFS	0.92	0.68	1.24	0.5845
			Y	Non-selective	PFS	1.67	0.99	2.79	0.0523
Jansen, 2017 (n=8100)	Colorectal	Any stage	N	Selective	OS	1.07	0.96	1.19	0.2412
			N	Non-selective	OS	1.01	0.84	1.22	0.9058
Johannesdottir, 2013 (n=6626)	Ovarian	Any stage	N	Any	OS	1.17	1.02	1.34	
Katsarelias, 2020 (n=12738)	Melanoma	Early/Non-metastatic	Y	Any	CSS	1	0.88	1.14	0.98
			Y	Non-selective	CSS	0.76	0.5	1.16	0.21
			Y	Selective	CSS	0.97	0.84	1.12	0.61
			Y	Non-selective	OS	0.94	0.76	1.16	0.55
			Y	Selective	OS	1.28	1.19	1.38	<0.001
Kennedy, 2022 (n=1019)	Melanoma	Advanced/Metastatic	Y	Any	PFS	0.96	0.7	1.31	0.793
Kim, 2017 (n=1274)	Head & Neck	Early/Non-metastatic	N	Any	CSS	1.81	1.20	2.72	0.004
			N	Any	OS	1.65	1.23	2.20	0.001
Kocak, 2023 (n=181)	Colorectal	Advanced/Metastatic	Y	Any	OS	0.57	0.36	0.91	0.02
			Y	Any	PFS	0.66	0.46	0.93	0.018
Kreklau, 2021 (n=635)	Breast	Any stage	N	Any	OS	0.27	0.12	0.6	0.01
			N	Any	PFS	0.53	0.27	1.04	0.07
Le Bozec, 2023 (n=182)	Pancreatic	Advanced/Metastatic	N	Any	OS	1.02	0.1	1.45	0.98
			N	Any	PFS	0.27	0.06	1.12	0.07
			N	Non-selective	OS	0.77	0.31	1.94	0.58
			N	Selective	OS	1.8	1.16	2.8	<0.01
Lemeshow, 2011 (n=4179)	Melanoma	Any stage	N	Any	CSS	0.87	0.64	1.2	0.408
			N	Any	CSS	0.36	0.2	0.66	0.001
			N	Any	OS	0.81	0.67	0.97	0.02
			N	Any	OS	0.78	0.6	1	0.052
Livingstone, 2013 (n=709)	Melanoma	Any stage	N	Any	OS	0.82	0.55	1.24	
Melhem-Bertrandt, 2011 (n=1413)	Breast	Any stage	Y	Any	OS	0.64	0.38	1.07	0.09
			Y	Any	PFS	0.52	0.31	0.88	0.015
Mellgard, 2023	Urothelial	Advanced/Metastatic	Y	Any	OS	0.24	0.09	0.62	0.003

(n=339)	Hepatocellular	Advanced/Metastatic	Y	Any	OS	1.03	0.48	2.24	0.94
	Melanoma	Advanced/Metastatic	Y	Any	OS	0.89	0.41	1.96	0.77
	Lung	Advanced/Metastatic	Y	Any	OS	0.82	0.4	1.65	0.57
Nayan, 2018 (n=9214)	Renal	Any stage	N	Any	CSS	1.02	0.90	1.16	
			N	Any	OS	0.75	0.70	0.81	
Oh, 2020 (n=109)	Lung	Advanced/Metastatic	Y	Any	PFS	0.48	0.23	1.01	0.054
Posielski, 2021 (n=39138)	Prostate	Advanced/Metastatic	Y	Any	CSS	0.94	0.87	1.03	0.17
			Y	Any	OS	0.97	0.93	1.01	0.14
Powe, 2010 (n=417)	Breast	Early/Non-metastatic	N	Any	CSS	0.291	0.119	0.715	0.007
Sakellakis, 2015 (n=610)	Breast	Early/Non-metastatic	NA	Any	PFS	0.849	0.537	1.343	0.485
Sanni, 2017 (n=3058)	Endometrial	Any stage	N	Any	CSS	0.87	0.68	1.10	0.24
			N	Any	OS	1.04	0.89	1.22	0.60
Santala, 2021 (n=9913)	Ovarian	Any stage	N	Any	CSS	1.02	0.95	1.09	
Scott, 2022 (n=14976)	Breast	Any stage	Y	Any	CSS	0.9	0.77	1.05	
Shah, 2011 (n=3462)	Breast	Any stage	N	Any	OS	1.09	0.8	1.49	
	Colorectal	Any stage	N	Any	OS	1	0.77	1.3	
	Lung	Any stage	N	Any	OS	1.12	0.89	1.41	
	Upper GI	Any stage	N	Any	OS	1.05	0.69	1.6	
	Ovarian	Any stage	N	Any	OS	1.14	0.63	2.06	
	Pancreatic	Any stage	N	Any	OS	1.88	1.09	3.25	
	Prostate	Any stage	N	Any	OS	1.54	1.13	2.09	
	Renal	Any stage	N	Any	OS	1.14	0.52	2.52	
	Upper GI	Any stage	N	Any	OS	1.44	0.76	2.74	
Siltari, 2020 (n=8253)	Prostate	Any stage	N	Any	CSS	1.18	1	1.39	
			N	Any	OS	1.22	1.12	1.33	
Springate, 2015 (n=11302)	Breast	Any stage	N	Any	OS	1.19	1.03	1.37	
	Colorectal	Any stage	N	Any	OS	0.85	0.74	0.97	
	Lung	Any stage	N	Any	OS	1.04	0.91	1.19	
	Upper GI	Any stage	N	Any	OS	1.27	1.01	1.59	
	Ovarian	Any stage	N	Any	OS	1.05	0.74	1.50	
	Pancreatic	Any stage	N	Any	OS	0.94	0.74	1.21	
	Prostate	Any stage	N	Any	OS	1.03	0.92	1.15	
	Renal	Any stage	N	Any	OS	0.46	0.26	0.83	
	Upper GI	Any stage	N	Any	OS	1.03	0.78	1.36	
Stoer, 2021 (n=2614)	Pancreatic	Any stage	N	Any	CSS	1.01	0.89	1.15	
Sud, 2018	Colorectal	Advanced/Metastatic	N	Any	PFS	0.72	0.51	1.03	0.077

(n=572)									
Tan, 2023 (n=2758)	Breast	Any stage	N	Any	CSS	0.86	0.58	1.28	
			N	Non-selective	CSS	0.42	0.14	1.25	
			N	Selective	CSS	0.95	0.63	1.43	
			N	Any	OS	1.02	0.79	1.32	
			N	Non-selective	OS	0.8	0.45	1.42	
			N	Selective	OS	1.07	0.81	1.4	
Udumyan, 2017 (n=2394)	Pancreatic	Any stage	N	Any	CSS	0.79	0.70	0.90	
			N	Selective	CSS	0.84	0.73	0.95	
			N	Non-selective	CSS	0.74	0.54	1.02	
Udumyan, 2020 (n=2104)	Hepatocellular	Any stage	N	Any	CSS	0.82	0.72	0.94	0.005
			N	Selective	CSS	0.86	0.75	1.00	0.049
Udumyan, 2020* (n=18429)	Lung	Any stage	N	Any	CSS	1.01	0.97	1.06	
			N	Non-selective	CSS	1.08	0.95	1.23	
			N	Selective	CSS	1.01	0.96	1.05	
Udumyan, 2022 (n=16669)	Urothelial	Any stage	N	Any	CSS	0.88	0.81	0.96	0.005
			N	Non-selective	CSS	0.66	0.5	0.86	0.002
			N	Selective	CSS	0.91	0.83	0.99	0.026
		Early/non-metastatic	N	Non-selective	CSS	1.06	0.71	1.6	0.762
			N	Selective	CSS	0.94	0.8	1.09	0.396
			N	Non-selective	OS	1.14	0.94	1.39	0.185
			N	Selective	OS	0.9	0.83	0.98	0.013
		Advanced/Metastatic	N	Non-selective	CSS	0.35	0.18	0.68	0.002
			N	Selective	CSS	0.97	0.78	1.2	0.757
			N	Non-selective	OS	0.45	0.26	0.78	0.004
			N	Selective	OS	0.94	0.77	1.15	0.567
Wang, 2015 (n=673)	Lung	Advanced/Metastatic	N	Any	OS	0.78	0.63	0.97	0.03
			N	Any	PFS	0.69	0.53	0.90	<0.01
Wang, 2019 (n=330)	Melanoma	Advanced/Metastatic	Y	Any	OS	0.89	0.6	1.32	0.57
			Y	Any	PFS	0.86	0.62	1.2	0.4
Watkins, 2015 (n=1425)	Ovarian	Any stage	Y	Any	CSS	1.82	1.39	2.38	<0.0001
			Y	Selective	CSS	2.05	1.51	2.79	<0.0001

			Y	Non-selective	CSS	2.48	1.25	4.93	<0.0001
			Y	Any	OS	1.70	1.31	2.19	<0.0001
			Y	Selective	OS	1.87	1.40	2.50	<0.0001
			Y	Non-selective	OS	2.47	1.25	4.91	0.010
Weberpals, 2017 (n=9876)	Prostate	Any stage	N	Any	OS	1.13	1.00	1.28	
	Colorectal	Any stage	N	Any	OS	1.15	1.05	1.26	
Weberpals, 2017* (n=3340)	Lung	Any stage	N	Any	OS	1.03	0.94	1.11	0.5509
			N	Selective	OS	0.99	0.91	1.08	0.8902
			N	Non-selective	OS	1.26	1.06	1.49	0.0071
Wrobel, 2020 (n=286)	Melanoma	Any stage	N	Any	CSS	0.25	0.06	1.13	0.071
			N	Any	OS	0.93	0.54	1.6	0.782
			N	Any	PFS	0.4	0.16	0.97	0.042
Wu, 2023 (n=578)	Hepatocellular	Advanced/Metastatic	Y	Any	PFS	1.02	0.83	1.26	0.844
			Y	Non-selective	PFS	0.92	0.66	1.29	0.629
			Y	Any	OS	1.12	0.9	1.39	0.298
			Y	Non-selective	OS	0.94	0.66	1.33	0.721
Yang, 2017 (n=606)	Lung	Advanced/Metastatic	N	Any	OS	0.825	0.630	2.333	0.564
Yang, 2021 (n=7130)	Pancreatic	Any stage	N	Any	OS	1.04	0.98	1.11	0.18
			N	Selective	OS	1.02	0.96	1.09	0.51
			N	Non-selective	OS	1.07	0.97	1.19	0.16
Zaborowska-Szmit, 2023 (n=196)	Lung	Advanced/Metastatic	Y	Any	OS	0.79	0.54	1.14	0.201
Zhang, 2022 (n=2343)	Colorectal	Any stage	N	Any	CSS	0.57	0.28	1.16	

S1. Search Strategy

Database: Ovid MEDLINE(R) and Epub Ahead of Print, In-Process & Other Non-Indexed Citations, Daily and Versions(R) <1946 to May 29, 2020>

Search Strategy:

----

1 exp Neoplasms/ (3322719)
2 (cancer* or neoplasm*).tw,kf. (1983238)
3 or/1-2 (3842495)
4 Neoplasm Recurrence, Local/ (116824)
5 exp Recurrence/ (182564)
6 recurren*.mp. (679308)
7 exp Survival/ (4707)
8 survival.mp. (1229363)
9 mortalit*.mp. (1142629)
10 relapse*.mp. (160010)
11 disease* free interval*.mp. (4102)
12 (metastas* adj1 develop*).mp. (4625)
13 (risk adj1 metastas*).mp. (375)
14 or/4-13 (2577203)
15 exp Acebutolol/ (844)
16 Acebutolol.mp. (1121)
17 Sectral.mp. (36)
18 exp Atenolol/ (5154)
19 Atenolol.mp. (8211)
20 Tenormin.mp. (73)
21 exp Bisoprolol/ (1075)
22 Bisoprolol.mp. (1717)
23 Zebeta.mp. (1)
24 exp Metoprolol/ (5437)
25 Metoprolol.mp. (8244)
26 Lopressor.mp. (23)
27 Toprol XL.mp. (5)
28 exp Nadolol/ (802)
29 Nadolol.mp. (1325)
30 Corgard.mp. (23)
31 exp Nebivolol/ (790)
32 Nebivolol.mp. (1042)
33 Bystolic.mp. (7)
34 exp Propranolol/ (32271)
35 Propranolol.mp. (44973)
36 Inderal.mp. (405)
37 InnoPran XL.mp. (2)
38 exp Betaxolol/ (651)
39 Betaxolol.mp. (978)
40 Kerlone.mp. (7)
41 exp Carvedilol/ (2620)
42 Carvedilol.mp. (3646)

43 Coreg.mp. (32)
44 exp Sotalol/ (2059)
45 Sotalol.mp. (3252)
46 Betapace.mp. (7)
47 Sorine.mp. (7)
48 Sotylize.mp. (0)
49 exp Timolol/ (3646)
50 Timolol.mp. (5033)
51 Blocadren.mp. (14)
52 exp Labetalol/ (1825)
53 Laetalol.mp. (0)
54 Normodyne.mp. (2)
55 Trandate.mp. (26)
56 exp Pindolol/ (3695)
57 Pindolol.mp. (4685)
58 Visken.mp. (143)
59 exp Adrenergic beta-Antagonists/ (83946)
60 beta blocker*.mp. (30441)
61 or/15-60 (113189)
62 3 and 14 and 61 (704)
63 limit 62 to english language (652)

Database: Embase Classic+Embase <1947 to 2020 Week 22>
Search Strategy:
----
1 malignant neoplasm/ (48040)
2 (cancer* or neoplasm*).tw,kw. (2818184)
3 or/1-2 (2832621)
4 exp cancer recurrence/ (181094)
5 recurren*.mp. (1038255)
6 exp cancer survival/ (367987)
7 survival.mp. (1737904)
8 exp cancer mortality/ (88869)
9 mortalit*.mp. (1531984)
10 relapse*.mp. (340721)
11 exp disease free interval/ (6549)
12 disease* free interval*.mp. (9850)
13 (metastas* adj1 develop*).mp. (7416)
14 (risk adj1 metastas*).mp. (593)
15 or/4-14 (3830070)
16 exp Acebutolol/ (5122)
17 Acebutolol.mp. (5198)
18 Sectral.mp. (680)
19 exp Atenolol/ (31211)
20 Atenolol.mp. (32103)
21 Tenormin.mp. (1662)
22 exp Bisoprolol/ (9546)

23 Bisoprolol.mp. (10216)
24 Zebeta.mp. (103)
25 exp Metoprolol/ (32993)
26 Metoprolol.mp. (36801)
27 Lopressor.mp. (843)
28 Toprol XL.mp. (209)
29 exp Nadolol/ (5548)
30 Nadolol.mp. (5675)
31 Corgard.mp. (499)
32 exp Nebivolol/ (3886)
33 Nebivolol.mp. (3967)
34 Bystolic.mp. (36)
35 exp Propranolol/ (98623)
36 Propranolol.mp. (104195)
37 Inderal.mp. (4638)
38 InnoPran XL.mp. (31)
39 exp Betaxolol/ (3264)
40 Betaxolol.mp. (3338)
41 Kerlone.mp. (143)
42 exp Carvedilol/ (15171)
43 Carvedilol.mp. (15406)
44 Coreg.mp. (314)
45 exp Sotalol/ (13264)
46 Sotalol.mp. (13569)
47 Betapace.mp. (168)
48 Sorine.mp. (23)
49 Sotylize.mp. (2)
50 exp Timolol/ (11503)
51 Timolol.mp. (14911)
52 Timolol.mp. (14911)
53 Blocadren.mp. (419)
54 exp Labetalol/ (10704)
55 Laetalol.mp. (0)
56 Normodyne.mp. (186)
57 Trandate.mp. (681)
58 exp Pindolol/ (10494)
59 Pindolol.mp. (10811)
60 Visken.mp. (1507)
61 exp Adrenergic beta-Antagonists/ (303674)
62 beta blocker*.mp. (49577)
63 or/16-62 (314056)
64 3 and 15 and 63 (1968)
65 limit 64 to english language (1902)

Database: EBM Reviews - Cochrane Central Register of Controlled Trials <April 2020>
Search Strategy:
----

1 exp Neoplasms/ (77335)
2 [(cancer* or neoplasm*).tw,kf.] (0)
3 or/1-2 (77335)
4 Neoplasm Recurrence, Local/ (4046)
5 exp Recurrence/ (11755)
6 recurren*.mp. (72187)
7 exp Survival/ (132)
8 survival.mp. (108716)
9 mortalit*.mp. (78486)
10 relapse*.mp. (38242)
11 disease* free interval*.mp. (1121)
12 (metastas* adj1 develop*).mp. (248)
13 (risk adj1 metastas*).mp. (167)
14 or/4-13 (231950)
15 exp Acebutolol/ (212)
16 Acebutolol.mp. (349)
17 Sectral.mp. (9)
18 exp Atenolol/ (1800)
19 Atenolol.mp. (3251)
20 Tenormin.mp. (47)
21 exp Bisoprolol/ (364)
22 Bisoprolol.mp. (985)
23 Zebeta.mp. (2)
24 exp Metoprolol/ (1615)
25 Metoprolol.mp. (3126)
26 Lopressor.mp. (6)
27 Toprol XL.mp. (24)
28 exp Nadolol/ (182)
29 Nadolol.mp. (375)
30 Corgard.mp. (4)
31 exp Nebivolol/ (0)
32 Nebivolol.mp. (555)
33 Bystolic.mp. (6)
34 exp Propranolol/ (2839)
35 Propranolol.mp. (5118)
36 Inderal.mp. (95)
37 InnoPran XL.mp. (1)
38 exp Betaxolol/ (199)
39 Betaxolol.mp. (375)
40 Kerlone.mp. (3)
41 exp Carvedilol/ (0)
42 Carvedilol.mp. (1396)
43 Coreg.mp. (34)
44 exp Sotalol/ (301)
45 Sotalol.mp. (618)
46 Betapace.mp. (0)
47 Sorine.mp. (0)
48 Sotylize.mp. (0)

49 exp Timolol/ (1180)
50 Timolol.mp. (2286)
51 Blocadren.mp. (10)
52 exp Labetalol/ (406)
53 Laetalol.mp. (0)
54 Normodyne.mp. (0)
55 Trandate.mp. (12)
56 exp Pindolol/ (519)
57 Pindolol.mp. (842)
58 Visken.mp. (45)
59 exp Adrenergic beta-Antagonists/ (10676)
60 beta blocker*.mp. (7123)
61 beta adrenergic receptor* blocking agent*.mp. (2624)
62 or/15-61 (22752)
63 3 and 14 and 62 (38)
64 limit 63 to english language (17)

Table S2. ROBINS-I Results

**First Author, Year of Publication**	Bias due to confounding	Bias in selection of participants into the study	Bias in classification of interventions	Bias due to deviations from intended interventions	Bias due to missing data	Bias in measurement of outcomes	Bias in selection of the reported result	Overall Bias
Altshuler, 2022	Serious	Low	Low	Moderate	Moderate	Low	Low	Serious
Assayag, 2014	Moderate	Low	Low	Low	Moderate	Low	Low	Moderate
Aydiner, 2013	Moderate	Low	Low	Low	NI	Low	Low	Moderate
Baek, 2018	Moderate	Moderate	Low	Low	Low	Low	Low	Moderate
Balkrishnan, 2021	Moderate	Low	Low	Low	Low	Low	Moderate	Moderate
Bar, 2016	Moderate	Low	Low	Low	Low	Low	Low	Moderate
Barron, 2011	Moderate	Low	Low	Critical	Low	Low	Low	Critical
Beg, 2018	Moderate	Moderate	Low	Low	Low	Low	Low	Moderate
Cardwell, 2013	Moderate	Low	Low	Low	Low	Low	Low	Moderate
Cardwell, 2016	Moderate	Low	Low	Low	Low	Low	Moderate	Moderate
Chang, 2019	Moderate	Serious	Moderate	Moderate	Moderate	Low	Low	Serious
Chang, 2020	Moderate	Low	Low	Low	Low	Low	Low	Moderate
Chen, 2017	Serious	Low	Low	Moderate	Low	Low	Low	Serious
Chen, 2023	Moderate	Low	Low	Moderate	Moderate	Low	Low	Moderate
Cho, 2020	Moderate	Moderate	Low	Low	Low	Low	Low	Moderate
Cortellini, 2021	Serious	Low	Low	Low	Low	Low	Low	Serious
Couttenier, 2019	Moderate	Moderate	Low	Low	NI	Low	Low	Moderate
Cui, 2019	Moderate	Moderate	Low	Low	Moderate	Low	Low	Moderate
De Giorgi, 2013	Serious	Low	Low	Low	Low	Low	Low	Serious
De Giorgi, 2018	Critical	Serious	Low	Moderate	Low	Low	Low	Critical
Eskelinen, 2022	Moderate	Low	Moderate	Serious	Moderate	Low	Serious	Serious
Failing, 2016	Serious	Low	Low	Moderate	Low	Low	Moderate	Serious
Farrugia, 2020	Serious	Low	Low	Low	Low	Low	Low	Serious
Fiala 2021	Serious	Low	Low	Low	Low	Low	Low	Serious
Fiala, 2019	Moderate	Moderate	Low	Low	NI	Low	Moderate	Moderate
Ganz, 2011	Serious	Low	Low	Moderate	NI	Low	Low	Serious
Giampieri, 2015	Serious	Low	Low	Low	NI	Low	Moderate	Serious
Gillis, 2021	Serious	Low	Low	Low	Low	Low	Low	Serious
Grytli, 2014	Serious	Low	Low	Low	Low	Low	Low	Serious
Hanley, 2021	Moderate	Low	Low	Low	Low	Low	Low	Moderate
Harding, 2019	Moderate	Moderate	Low	Low	Low	Low	Low	Moderate
He, 2015	Moderate	Low	Low	Low	Low	Low	Moderate	Moderate

Heitz, 2013	Serious	Serious	Low	NI	NI	Low	Low	Serious
Hicks, 2013	Moderate	Low	Low	Low	Moderate	Low	Low	Moderate
Holmes, 2013	Serious	Low	Low	Low	NI	Low	Low	Serious
Hsieh, 2023	Moderate	Low	Low	Moderate	Critical	Low	Low	Critical
Huang, 2021	Serious	Low	Low	Low	Low	Low	Low	Serious
Jansen, 2014	Moderate	Moderate	Low	Low	Low	Low	Low	Moderate
Jansen, 2017	Moderate	Low	Low	Low	NI	Low	Low	Moderate
Johannesdottir, 2013	Moderate	Low	Low	Low	Low	Low	Low	Moderate
Katsarelias, 2020	Serious	Low	Low	Low	Low	Low	Low	Serious
Kennedy, 2022	Serious	Low	Low	Low	Low	Low	Low	Serious
Kim, 2017	Moderate	Moderate	Low	Low	NI	Low	Low	Moderate
Kocak, 2023	Moderate	Serious	Low	Moderate	Moderate	Low	Low	Serious
Kreklau, 2021	Serious	Moderate	Serious	Low	Low	Low	Serious	Serious
Le Bozec, 2023	Moderate	Low	Moderate	Moderate	Low	Low	Low	Moderate
Lemeshow, 2011	Moderate	Low	Low	Low	Low	Low	Low	Moderate
Livingstone, 2013	Moderate	Low	Low	NI	Low	Low	Low	Moderate
Melhem-Bertrand, 2011	Serious	Low	Low	Low	Low	Low	Low	Serious
Mellgard, 2023	Serious	Low	Low	Serious	Moderate	Low	Low	Serious
Nayan, 2018	Moderate	Moderate	Low	Low	Low	Low	Low	Moderate
Oh, 2020	Serious	Low	Low	Low	Low	Low	Low	Serious
Posielski, 2021	Moderate	Low	Low	Low	Low	Low	Serious	Serious
Powe, 2010	Serious	Low	Low	Moderate	NI	Low	Low	Serious
Sakellakis, 2015	Serious	Moderate	Serious	Low	NI	Low	Moderate	Serious
Sanni, 2017	Moderate	Low	Low	Low	Moderate	Low	Low	Moderate
Santala, 2021	Serious	Low	Low	Low	Low	Low	Low	Serious
Scott, 2022	Moderate	Low	Low	Low	Low	Low	Low	Moderate
Shah, 2011	Serious	Low	Low	Moderate	Moderate	Low	Low	Serious
Siltari, 2020	Moderate	Low	Low	Low	Low	Low	Low	Moderate
Springate, 2015	Moderate	Low	Low	Low	Low	Low	Moderate	Moderate
Stoer, 2021	Moderate	Low	Low	Low	Low	Low	Low	Moderate
Sud, 2018	Serious	Serious	Serious	Low	NI	Low	Low	Serious
Tan, 2023	Moderate	Serious	Low	Serious	Critical	Low	Low	Critical
Udumyan, 2017	Serious	Low	Low	Low	Low	Low	Low	Serious
Udumyan, 2020	Moderate	Moderate	Low	Low	NI	Low	Low	Moderate
Udumyan, 2020	Moderate	Moderate	Low	Low	NI	Low	Low	Moderate

Udumyan, 2022	Low	Low	Low	Moderate	Low	Low	Low	Moderate
Wang, 2015	Critical	Moderate	Low	Critical	Low	Low	Low	Critical
Wang, 2019	Serious	Low	Low	Low	Low	Low	Moderate	Serious
Watkins, 2015	Serious	Low	Low	Low	Low	Low	Moderate	Serious
Weberpals, 2017	Moderate	Moderate	Low	Low	Moderate	Low	Low	Moderate
Weberpals, 2017	Moderate	Low	Low	Low	Moderate	Low	Moderate	Moderate
Wrobel, 2020	Serious	NI	Serious	Low	Low	Low	Low	Serious
Wu, 2023	Serious	Low	Low	Moderate	Moderate	Low	Low	Serious
Yang, 2017	Serious	Serious	Serious	Low	Low	Low	Low	Serious
Yang, 2021	Moderate	Low	Low	Low	Low	Low	Low	Moderate
Zaborowska-Szmit, 2023	Serious	Low	Moderate	Moderate	Moderate	Low	Low	Serious
Zhang, 2022	Moderate	Low	Low	Moderate	Low	Low	Moderate	Moderate

Table S3. Actively recruiting and not yet recruiting trials in the ClinicalTrials.gov database

ClinicalTrials.gov Identifier	Start date	Country	Beta blocker type	Primary Outcome(s)	Phase	Included primary cancer type(s)	Included cancer stage(s)
NCT03384836	January 31, 2018	United States	Propranolol hydrochloride	Dose limiting toxicities, overall response rate	Ib/II	Melanoma	Unresectable stage III and IV
NCT04493489	September 6, 2020	China	Propranolol hydrochloride	Two year recurrence free survival	II	Bladder	T1/Ta high grade/CIS
NCT04682158	April 1, 2021	United States	Propranolol	Occurrence of adverse events, Progression free survival	II	Esophageal	Any
NCT04848519	May 20, 2021	United States	Propranolol hydrochloride	Incidence of adverse events	II	Urothelial	Locally advanced or metastatic
NCT05961761	August 17, 2021	Denmark	Propranolol	Progression-free survival rate	II	Soft tissue sarcoma	Unresectable locally advanced or metastatic
NCT05451043	March 1, 2023	Canada	Propranolol	Objective response rate	II	Hepatopancreatobiliary	Advanced
NCT05651594	March 7, 2023	United States	Propranolol	Overall response rate	II	Esophageal or Gastroesophageal	Unresectable locally advanced
NCT05968690	September 11, 2023	United States	Propranolol	Safety, dose-limiting toxicity, recommended phase 2 dose	I	Melanoma	Unresectable stage III and IV
NCT05979818	December 31, 2023	China	Propranolol hydrochloride	Objective response rate, disease control rate, adverse events	I	Lung	IIIB-IIIC, IV
NCT05741164	April 15, 2024	United States	Propranolol	Objective response	II	Breast	Metastatic or unresectable

Table S4. ROBINS-I Summary

Risk of bias comparison by outcome					
	**All studies (n=79)**	**OS (n=56)**	**PFS (n=27)**	**CSS (n=37)**	**p-value**
**Confounding**					0.1465
Low	n=1 (1.3%)	n=1 (1.8%)	n=0 (0%)	n=1 (2.7%)	
Moderate	n=44 (5.1%)	n=32 (57.1%)	n=8 (29.6%)	n=25 (67.6%)	
Serious	n=32 (40.5%)	n=21 (37.5%)	n=17 (63.0%)	n=11 (29.7%)	
Critical	n=2 (2.5%)	n=2 (3.6%)	n=2 (7.4%)	n=0 (0%)	
NI	n=0 (0%)	n=0 (0%)	n=0 (0%)	n=0 (0%)	
**Selection of Participants**					0.8945
Low	n=55 (69.6%)	n=38 (67.9%)	n=16 (59.3%)	n=27 (73.0%)	
Moderate	n=16 (20.2%)	n=11 (19.6%)	n=6 (22.2%)	n=8 (21.6%)	
Serious	n=7 (88.6%)	n=6 (10.7%)	n=4 (14.8%)	n=1 (2.7%)	
Critical	n=1 (1.3%)	n=0 (0%)	n=0 (0%)	n=0 (0%)	
NI	n=0 (0%)	n=1 (1.8%)	n=1 (3.7%)	n=1 (2.7%)	
**Classification**					0.8194
Low	n=56 (70.9%)	n=50 (89.3%)	n=22 (81.5%)	n=35 (94.6%)	
Moderate	n=16 (20.2%)	n=3 (5.3%)	n=1 (3.7%)	n=1 (2.7%)	
Serious	n=3 (3.8%)	n=3 (5.3%)	n=4 (14.8%)	n=1 (2.7%)	
Critical	n=2 (2.5%)	n=0 (0%)	n=0 (0%)	n=0 (0%)	
NI	n=2 (2.5%)	n=0 (0%)	n=0 (0%)	n=0 (0%)	
**Deviations**					0.7928
Low	n=56 (70.9%)	n=38 (67.9%)	n=17 (63.0%)	n=28 (75.7%)	
Moderate	n=16 (20.2%)	n=13 (23.2%)	n=8 (29.6%)	n=6 (16.2%)	
Serious	n=3 (3.8%)	n=2 (3.6%)	n=0 (0%)	n=2 (5.4%)	
Critical	n=2 (2.5%)	n=1 (1.8%)	n=1 (3.7%)	n=1 (2.7%)	
NI	n=2 (2.5%)	n=2 (3.6%)	n=1 (3.7%)	n=0 (0%)	
**Missing data**					0.9956
Low	n=48 (60.8%)	n=32 (57.1%)	n=17 (63.0%)	n=24 (64.9%)	
Moderate	n=15 (19.0%)	n=13 (23.2%)	n=4 (14.8%)	n=6 (16.2%)	
Serious	n=0 (0%)	n=0 (0%)	n=0 (0%)	n=0 (0%)	
Critical	n=2 (2.5%)	n=2 (3.6%)	n=1 (3.7%)	n=1 (2.7%)	
NI	n=14 (17.7%)	n=9 (16.1%)	n=5 (18.5%)	n=6 (16.2%)	
**Measurement**					1
Low	n=79 (100%)	n=56 (100%)	n=27 (100%)	n=37 (100.0%)	
Moderate	n=0 (0%)	n=0 (0%)	n=0 (0%)	n=0 (0%)	
Serious	n=0 (0%)	n=0 (0%)	n=0 (0%)	n=0 (0%)	
Critical	n=0 (0%)	n=0 (0%)	n=0 (0%)	n=0 (0%)	
NI	n=0 (0%)	n=0 (0%)	n=0 (0%)	n=0 (0%)	

**Selection of Reported Result**					0.9999
Low	n=64 (81.0%)	n=45 (80.4%)	n=21 (77.8%)	n=30 (81.1%)	
Moderate	n=12 (15.2%)	n=9 (16.1%)	n=5 (18.5%)	n=5 (13.5%)	
Serious	n=3 (3.8%)	n=2 (3.6%)	n=1 (3.7%)	n=2 (5.4%)	
Critical	n=0 (0%)	n=0 (0%)	n=0 (0%)	n=0 (0%)	
NI	n=0 (0%)	n=0 (0%)	n=0 (0%)	n=0 (0%)	
**Overall Bias**					0.328
Low	n=0 (0%)	n=0 (0%)	n=0 (0%)	n=0 (0%)	
Moderate	n=38 (48.1%)	n=28 (50.0%)	n=6 (22.2%)	n=22 (59.4%)	
Serious	n=36 (45.6%)	n=24 (42.9%)	n=18 (66.7%)	n=13 (35.1%)	
Critical	n=5 (6.3%)	n=4 (71.4%)	n=3 (11.1%)	n=2 (5.4%)	
NI	n=0 (0%)	n=0 (0%)	n=0 (0%)	n=0 (0%)	

Table S5. PRISMA 2020 Checklist

Section and Topic	Item #	Checklist item	Location where item is reported
**TITLE**			

Section and Topic	Item #	Checklist item	Location where item is reported
Title	1	Identify the report as a systematic review.	Title page (1)
**ABSTRACT**			
Abstract	2	See the PRISMA 2020 for Abstracts checklist.	Page 1-2
**INTRODUCTION**			
Rationale	3	Describe the rationale for the review in the context of existing knowledge.	Page 3
Objectives	4	Provide an explicit statement of the objective(s) or question(s) the review addresses.	Page 3
**METHODS**			
Eligibility criteria	5	Specify the inclusion and exclusion criteria for the review and how studies were grouped for the syntheses.	Page 4
Information sources	6	Specify all databases, registers, websites, organisations, reference lists and other sources searched or consulted to identify studies. Specify the date when each source was last searched or consulted.	Page 4
Search strategy	7	Present the full search strategies for all databases, registers and websites, including any filters and limits used.	Supplement 1
Selection process	8	Specify the methods used to decide whether a study met the inclusion criteria of the review, including how many reviewers screened each record and each report retrieved, whether they worked independently, and if applicable, details of automation tools used in the process.	Page 4
Data collection process	9	Specify the methods used to collect data from reports, including how many reviewers collected data from each report, whether they worked independently, any processes for obtaining or confirming data from study investigators, and if applicable, details of automation tools used in the process.	Page 4-5
Data items	10a	List and define all outcomes for which data were sought. Specify whether all results that were compatible with each outcome domain in each study were sought (e.g. for all measures, time points, analyses), and if not, the methods used to decide which results to collect.	Page 4-5
	10b	List and define all other variables for which data were sought (e.g. participant and intervention characteristics, funding sources). Describe any assumptions made about any missing or unclear information.	Page 4-5
Study risk of bias assessment	11	Specify the methods used to assess risk of bias in the included studies, including details of the tool(s) used, how many reviewers assessed each study and whether they worked independently, and if applicable, details of automation tools used in the process.	Page 5
Effect measures	12	Specify for each outcome the effect measure(s) (e.g. risk ratio, mean difference) used in the synthesis or presentation of results.	Page 5
Synthesis methods	13a	Describe the processes used to decide which studies were eligible for each synthesis (e.g. tabulating the study intervention characteristics and comparing against the planned groups for each synthesis (item #5)).	Page 5
	13b	Describe any methods required to prepare the data for presentation or synthesis, such as handling of missing summary statistics, or data conversions.	Page 5
	13c	Describe any methods used to tabulate or visually display results of individual studies and syntheses.	N/A
	13d	Describe any methods used to synthesize results and provide a rationale for the choice(s). If meta-analysis was performed, describe the model(s), method(s) to identify the presence and extent of statistical heterogeneity, and software package(s) used.	Page 5
	13e	Describe any methods used to explore possible causes of heterogeneity among study results (e.g. subgroup analysis, meta-regression).	Page 5
	13f	Describe any sensitivity analyses conducted to assess robustness of the synthesized results.	Page 5
Reporting bias assessment	14	Describe any methods used to assess risk of bias due to missing results in a synthesis (arising from reporting biases).	Page 5

Section and Topic	Item #	Checklist item	Location where item is reported
Certainty assessment	15	Describe any methods used to assess certainty (or confidence) in the body of evidence for an outcome.	Page 5
**RESULTS**			
Study selection	16a	Describe the results of the search and selection process, from the number of records identified in the search to the number of studies included in the review, ideally using a flow diagram.	Page 5-6
	16b	Cite studies that might appear to meet the inclusion criteria, but which were excluded, and explain why they were excluded.	Figure 2
Study characteristics	17	Cite each included study and present its characteristics.	Supplemental Table 1
Risk of bias in studies	18	Present assessments of risk of bias for each included study.	Supplemental Table 2
Results of individual studies	19	For all outcomes, present, for each study: (a) summary statistics for each group (where appropriate) and (b) an effect estimate and its precision (e.g. confidence/credible interval), ideally using structured tables or plots.	Supplemental Table 1
Results of syntheses	20a	For each synthesis, briefly summarise the characteristics and risk of bias among contributing studies.	Page 10-11
	20b	Present results of all statistical syntheses conducted. If meta-analysis was done, present for each the summary estimate and its precision (e.g. confidence/credible interval) and measures of statistical heterogeneity. If comparing groups, describe the direction of the effect.	Page 6-11
	20c	Present results of all investigations of possible causes of heterogeneity among study results.	Page 6-7
	20d	Present results of all sensitivity analyses conducted to assess the robustness of the synthesized results.	Page 11
Reporting biases	21	Present assessments of risk of bias due to missing results (arising from reporting biases) for each synthesis assessed.	N/A
Certainty of evidence	22	Present assessments of certainty (or confidence) in the body of evidence for each outcome assessed.	Page 11, Supplemental Table 7
**DISCUSSION**			
Discussion	23a	Provide a general interpretation of the results in the context of other evidence.	Page 11-13
	23b	Discuss any limitations of the evidence included in the review.	Page 12
	23c	Discuss any limitations of the review processes used.	Page 12
	23d	Discuss implications of the results for practice, policy, and future research.	Page 12-13
**OTHER INFORMATION**			
Registration and protocol	24a	Provide registration information for the review, including register name and registration number, or state that the review was not registered.	Page 5
	24b	Indicate where the review protocol can be accessed, or state that a protocol was not prepared.	Page 5
	24c	Describe and explain any amendments to information provided at registration or in the protocol.	Page 5
Support	25	Describe sources of financial or non-financial support for the review, and the role of the funders or sponsors in the review.	Page 5
Competing interests	26	Declare any competing interests of review authors.	Page 13
Availability of data, code and other materials	27	Report which of the following are publicly available and where they can be found: template data collection forms; data extracted from included studies; data used for all analyses; analytic code; any other materials used in the review.	Page 13

From: Page MJ, McKenzie JE, Bossuyt PM, Boutron I, Hoffmann TC, Mulrow CD, et al. The PRISMA 2020 statement: an updated guideline for reporting systematic reviews. BMJ 2021;372:n71. doi: 10.1136/bmj.n71

Table S6. Pooled HRs

**OVERALL SURVIVAL**		
**Any beta-blocker**	**All studies: Pooled HR (95% CI)**	**ITB = 'N': Pooled HR (95% CI)**
All cancer types	76 studies: 0.99 (0.94-1.04)	54 studies: 1.00 (0.93-1.06)
Ovarian (n=9)	1.09 (0.94-1.26)	5 studies: **1.17 (1.11-1.25)**
Breast (n=9)	1.07 (0.86-1.32)	6 studies: 1.04 (0.86-1.25)
Colorectal (n=9)	0.90 (0.77-1.06)	7 studies: 0.93 (0.78-1.10)
Lung (n=12)	0.91 (0.78-1.05)	8 studies: 0.92 (0.77-1.10)
Upper GI (n=7)	0.95 (0.77-1.18)	6 studies: 1.04 (0.88-1.22)
Renal (n=3)	0.71 (0.50-1.02)	3 studies: 0.71 (0.50-1.02)
Prostate (n=8)	1.08 (0.98-1.18)	7 studies: 1.10 (0.99-1.22)
Head & Neck (n=2)	**1.66 (1.30-2.11)**	
Pancreatic (n=5)	1.00 (0.87-1.14)	4 studies: 1.07 (0.89-1.29)
Melanoma (n=8)	**0.81 (0.72-0.92)**	6 studies: **0.80 (0.68-0.95)**
Hepatocellular (n=2)	1.11 (0.90-1.37)	
**Non-selective beta-blocker**	**All studies: Pooled HR (95% CI)**	**ITB = 'N': Pooled HR (95% CI)**
All cancer types	19 studies: 1.04 (0.92-1.17)	13 studies: 1.01 (0.89-1.14)
**Selective beta-blocker**	**All studies: Pooled HR (95% CI)**	**ITB = 'N': Pooled HR (95% CI)**
All cancer types	13 studies: 1.11 (1.00-1.23)	9 studies: 1.01 (0.93-1.10)
**PROGRESSION-FREE SURVIVAL**		
**Any beta-blocker**	**All studies: Pooled HR (95% CI)**	**ITB = 'N': Pooled HR (95% CI)**
All cancer types	26 studies: **0.78 (0.66-0.92)**	8 studies: 0.74 (0.61-0.90)
Colorectal (n=5)	0.85 (0.69-1.04)	3 studies: 0.85 (0.64-1.12)
Melanoma (n=5)	**0.53 (0.28-0.98)**	
Breast (n=4)	0.87 (0.41-1.84)	
Renal (n=2)	**0.46 (0.35-0.60)**	
Lung (n=3)	0.77 (0.53-1.13)	
**Non-selective beta blocker**	**All studies: Pooled HR (95% CI)**	**ITB = 'N': Pooled HR (95% CI)**
All cancer types	5 studies: 1.19 (0.78-1.81)	3 studies: 1.00 (0.61-1.66)
**Selective beta blocker**	**All studies: Pooled HR (95% CI)**	**ITB = 'N': Pooled HR (95% CI)**
All cancer types	3 studies: 1.17 (0.55-2.52)	N/A
**CANCER-SPECIFIC SURVIVAL**		
**Any beta-blocker**	**All studies: Pooled HR (95% CI)**	**ITB = 'N': Pooled HR (95% CI)**
All cancer types	39 studies: 0.95 (0.91-1.00)	33 studies: 1.01 (0.88-1.02)
Lung (n=2)	1.01 (0.96-1.05)	2 studies: 1.01 (0.96-1.05)
Ovarian (n=5)	1.02 (0.93-1.13)	5 studies: 1.02 (0.93-1.13)
Breast (n=7)	0.94 (0.78-1.13)	5 studies: 0.94 (0.70-1.28)
Colorectal (n=5)	**0.83 (0.73-0.95)**	4 studies: **0.81 (0.69-0.94)**
Upper GI (n=2)	0.96 (0.74-1.24)	
Renal (n=3)	**1.08 (1.03-1.13)**	3 studies: **1.08 (1.03-1.13)**

Head & Neck (n=2)	**1.68 (1.23-2.28)**	
Pancreatic (n=2)	0.89 (0.70-1.14)	
Prostate (n=4)	0.95 (0.81-1.12)	3 studies: 0.96 (0.73-1.28)
Melanoma (n=4)	0.69 (0.44-1.06)	3 studies: 0.50 (0.23-1.09)
**Non-selective beta-blocker**	**All studies: Pooled HR (95% CI)**	**ITB = 'N': Pooled HR (95% CI)**
All cancer types	14 studies: 0.86 (0.73-1.00)	12 studies: 0.83 (0.70-1.00)
**Selective beta-blocker**	**All studies: Pooled HR (95% CI)**	**ITB = 'N': Pooled HR (95% CI)**
All cancer types	14 studies: 0.95 (0.89-1.01)	12 studies: 0.95 (0.88-1.02)

Figure S1. Subgroup Analysis of PFS by Cancer Stage

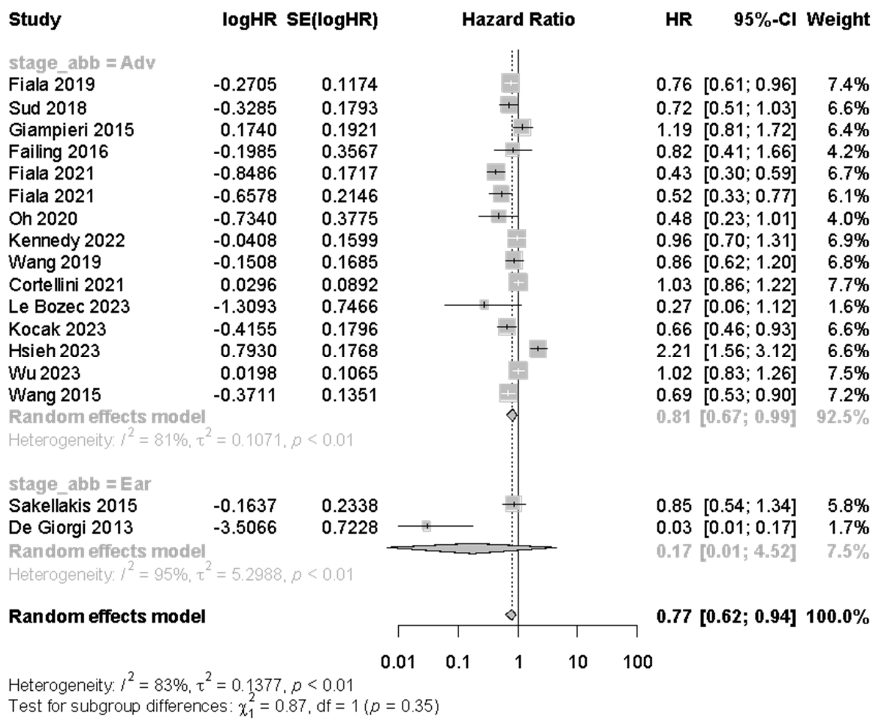

Figure S2. Subgroup Analysis of PFS by Non-selective vs Any Beta Blockers

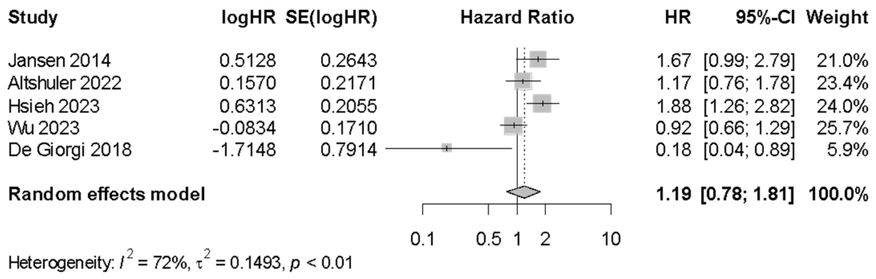

Figure S3. Subgroup Analysis of PFS by Selective vs Any Beta Blockers

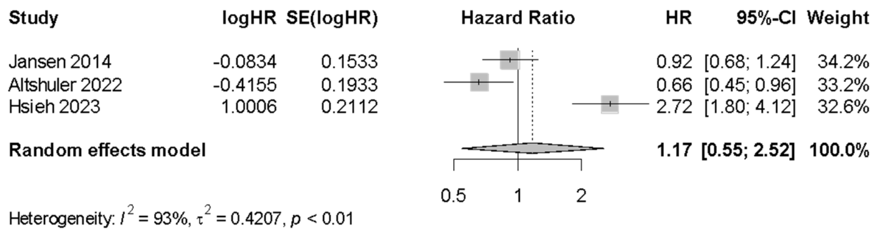

Figure S4. PFS ITB Sensitivity Analysis

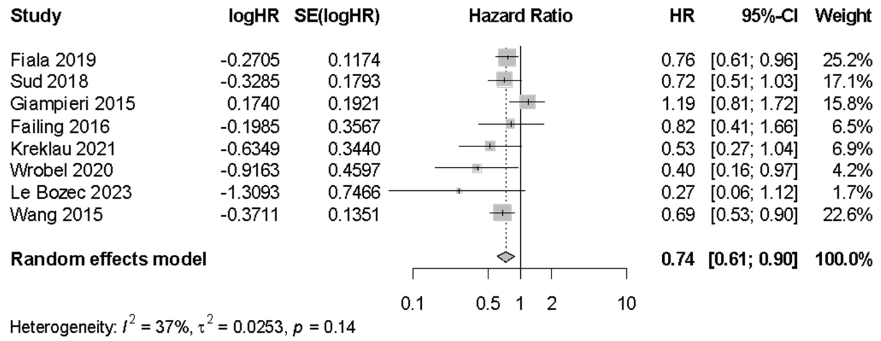

Figure S5. Subgroup Analysis of OS by Cancer Type

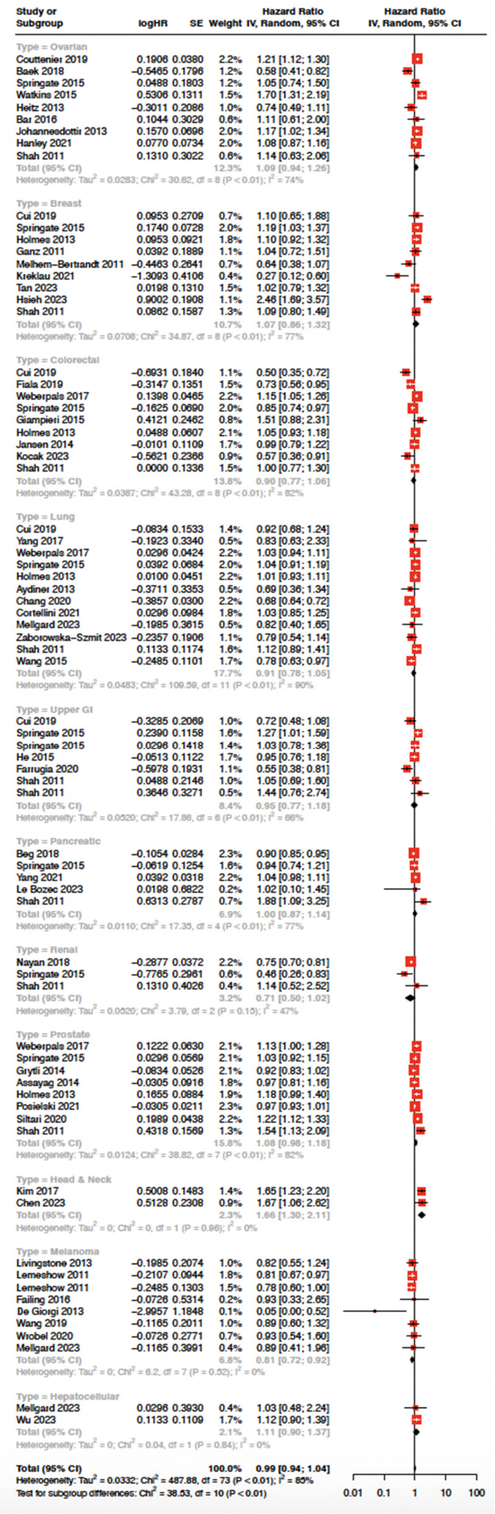

Figure S6. Subgroup Analysis of OS by Cancer Stage

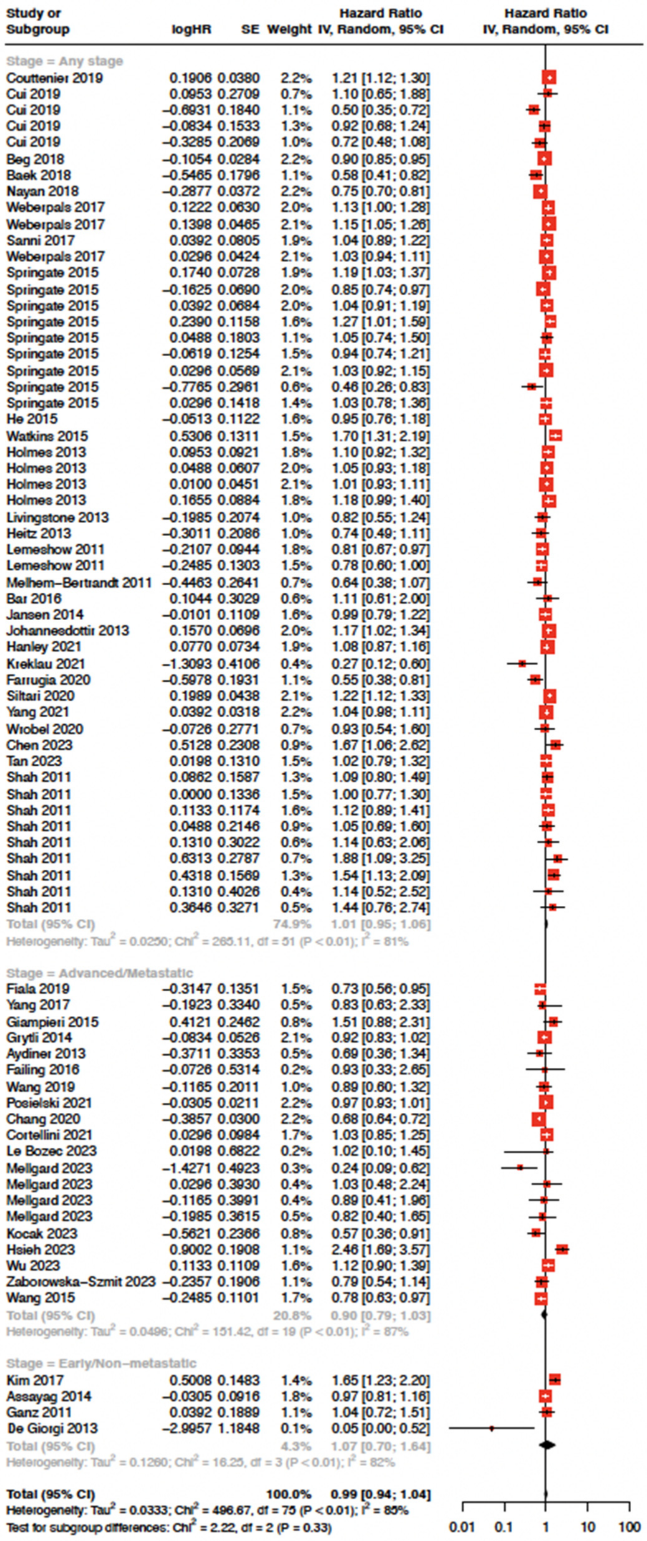

Figure S7. Subgroup Analysis of OS by Non-selective vs Any Beta Blocker Use

Study	logHR	SE	Weight	Hazard Ratio IV, Random, 95% CI
Couttenier 2019	0.1823	0.0574	7.8%	1.20 [1.07; 1.34]
Baek 2018	-0.5709	0.2068	4.2%	0.56 [0.38; 0.85]
Weberpals 2017	0.2311	0.0869	7.2%	1.26 [1.06; 1.49]
Jansen 2017	0.0100	0.0952	7.0%	1.01 [0.84; 1.22]
Cardwell 2016	0.1484	0.0658	7.6%	1.16 [1.02; 1.32]
Watkins 2015	0.9042	0.3490	2.2%	2.47 [1.25; 4.91]
Assayag 2014	-0.0619	0.1190	6.3%	0.94 [0.74; 1.18]
Jansen 2014	0.3507	0.1883	4.6%	1.42 [0.98; 2.05]
Katsarelias 2020	-0.0619	0.1079	6.6%	0.94 [0.76; 1.16]
Yang 2021	0.0677	0.0521	7.9%	1.07 [0.97; 1.19]
Le Bozec 2023	-0.2614	0.4678	1.4%	0.77 [0.31; 1.94]
Altshuler 2022	0.0488	0.0866	7.2%	1.05 [0.94; 1.32]
Udumyan 2022	0.1310	0.0998	6.8%	1.14 [0.94; 1.39]
Udumyan 2022	-0.7985	0.2803	3.0%	0.45 [0.26; 0.78]
Tan 2023	-0.2231	0.2932	2.8%	0.80 [0.45; 1.42]
Hsieh 2023	0.7419	0.2222	3.9%	2.10 [1.36; 3.25]
Wu 2023	-0.0619	0.1788	4.8%	0.94 [0.66; 1.33]
De Giorgi 2018	-0.4463	0.9385	0.4%	0.64 [0.10; 3.96]
Chang 2019	-0.2485	0.0393	8.1%	0.78 [0.72; 0.84]
**Total (95% CI)**			**100.0%**	**1.04 [0.92; 1.17]**

Heterogeneity: $Tau^2 = 0.0436$; $Chi^2 = 104.81$, df = 18 ($P < 0.01$); $I^2 = 83\%$

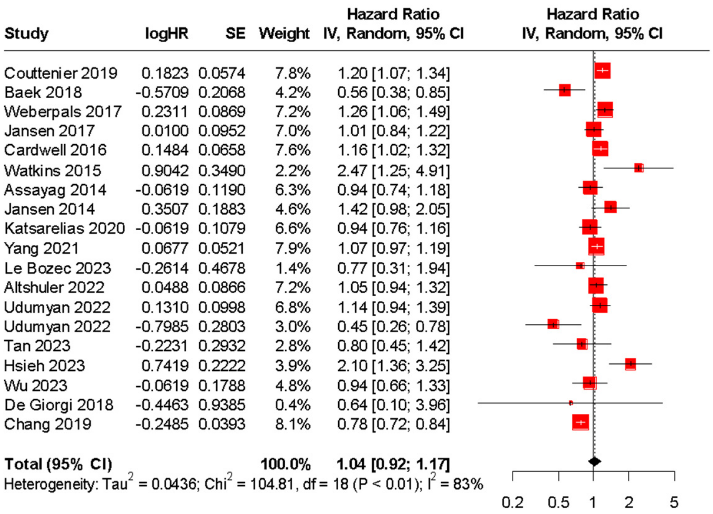

Figure S8. Subgroup Analysis of OS by Selective vs Any Beta Blocker Use

Study	logHR	SE	Weight	Hazard Ratio IV, Random, 95% CI
Couttenier 2019	0.1570	0.0413	9.9%	1.17 [1.08; 1.27]
Weberpals 2017	-0.0101	0.0437	9.8%	0.99 [0.91; 1.08]
Jansen 2017	0.0677	0.0548	9.4%	1.07 [0.96; 1.19]
Watkins 2015	0.6259	0.1479	5.8%	1.87 [1.40; 2.50]
Jansen 2014	-0.1278	0.1153	7.0%	0.88 [0.70; 1.10]
Katsarelias 2020	0.2469	0.0378	10.0%	1.28 [1.19; 1.38]
Yang 2021	0.0198	0.0324	10.1%	1.02 [0.96; 1.09]
Le Bozec 2023	0.5878	0.2248	3.6%	1.80 [1.16; 2.80]
Altshuler 2022	-0.2877	0.1103	7.2%	0.75 [0.61; 0.94]
Udumyan 2022	-0.1054	0.0424	9.8%	0.90 [0.83; 0.98]
Udumyan 2022	-0.0619	0.1023	7.6%	0.94 [0.77; 1.15]
Tan 2023	0.0677	0.1396	6.1%	1.07 [0.81; 1.40]
Hsieh 2023	1.0886	0.2253	3.6%	2.97 [1.91; 4.62]
**Total (95% CI)**			**100.0%**	**1.11 [1.00; 1.23]**

Heterogeneity: $Tau^2 = 0.0269$; $Chi^2 = 104.14$, df = 12 ($P < 0.01$); $I^2 = 88\%$

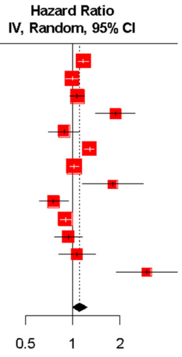

Figure S9. OS ITB Sensitivity Analysis

Study	logHR	SE	Weight	Hazard Ratio IV, Random, 95% CI
Couttenier 2019	0.1906	0.0380	2.7%	1.21 [1.12; 1.30]
Cui 2019	0.0953	0.2709	1.0%	1.10 [0.65; 1.88]
Cui 2019	–0.6931	0.1840	1.5%	0.50 [0.35; 0.72]
Cui 2019	–0.0834	0.1533	1.8%	0.92 [0.68; 1.24]
Cui 2019	–0.3285	0.2069	1.4%	0.72 [0.48; 1.08]
Fiala 2019	–0.3147	0.1351	1.9%	0.73 [0.56; 0.95]
Nayan 2018	–0.2877	0.0372	2.7%	0.75 [0.70; 0.81]
Weberpals 2017	0.1222	0.0630	2.6%	1.13 [1.00; 1.28]
Weberpals 2017	0.1398	0.0465	2.7%	1.15 [1.05; 1.26]
Kim 2017	0.5008	0.1483	1.8%	1.65 [1.23; 2.20]
Sanni 2017	0.0392	0.0805	2.4%	1.04 [0.89; 1.22]
Yang 2017	–0.1923	0.3340	0.7%	0.83 [0.63; 2.33]
Weberpals 2017	0.0296	0.0424	2.7%	1.03 [0.94; 1.11]
Springate 2015	0.1740	0.0728	2.5%	1.19 [1.03; 1.37]
Springate 2015	–0.1625	0.0690	2.5%	0.85 [0.74; 0.97]
Springate 2015	0.0392	0.0684	2.5%	1.04 [0.91; 1.19]
Springate 2015	0.2390	0.1158	2.1%	1.27 [1.01; 1.59]
Springate 2015	0.0488	0.1803	1.6%	1.05 [0.74; 1.50]
Springate 2015	–0.0619	0.1254	2.0%	0.94 [0.74; 1.21]
Springate 2015	0.0296	0.0569	2.6%	1.03 [0.92; 1.15]
Springate 2015	–0.7765	0.2961	0.9%	0.46 [0.26; 0.83]
Springate 2015	0.0296	0.1418	1.9%	1.03 [0.78; 1.36]
He 2015	–0.0513	0.1122	2.1%	0.95 [0.76; 1.18]
Giampieri 2015	0.4121	0.2462	1.1%	1.51 [0.88; 2.31]
Grytli 2014	–0.0834	0.0526	2.7%	0.92 [0.83; 1.02]
Assayag 2014	–0.0305	0.0916	2.3%	0.97 [0.81; 1.16]
Holmes 2013	0.0953	0.0921	2.3%	1.10 [0.92; 1.32]
Holmes 2013	0.0488	0.0607	2.6%	1.05 [0.93; 1.18]
Holmes 2013	0.0100	0.0451	2.7%	1.01 [0.93; 1.11]
Holmes 2013	0.1655	0.0884	2.4%	1.18 [0.99; 1.40]
Livingstone 2013	–0.1985	0.2074	1.4%	0.82 [0.55; 1.24]
Lemeshow 2011	–0.2107	0.0944	2.3%	0.81 [0.67; 0.97]
Lemeshow 2011	–0.2485	0.1303	2.0%	0.78 [0.60; 1.00]
Failing 2016	–0.0726	0.5314	0.3%	0.93 [0.33; 2.65]
De Giorgi 2013	–2.9957	1.1848	0.1%	0.05 [0.00; 0.52]
Johannesdottir 2013	0.1570	0.0696	2.5%	1.17 [1.02; 1.34]
Hanley 2021	0.0770	0.0734	2.5%	1.08 [0.87; 1.16]
Chang 2020	–0.3857	0.0300	2.8%	0.68 [0.64; 0.72]
Kreklau 2021	–1.3093	0.4106	0.5%	0.27 [0.12; 0.60]
Siltari 2020	0.1989	0.0438	2.7%	1.22 [1.12; 1.33]
Yang 2021	0.0392	0.0318	2.8%	1.04 [0.98; 1.11]
Wrobel 2020	–0.0726	0.2771	1.0%	0.93 [0.54; 1.60]
Le Bozec 2023	0.0198	0.6822	0.2%	1.02 [0.10; 1.45]
Tan 2023	0.0198	0.1310	2.0%	1.02 [0.79; 1.32]
Shah 2011	0.0862	0.1587	1.7%	1.09 [0.80; 1.49]
Shah 2011	0.0000	0.1336	1.9%	1.00 [0.77; 1.30]
Shah 2011	0.1133	0.1174	2.1%	1.12 [0.89; 1.41]
Shah 2011	0.0488	0.2146	1.3%	1.05 [0.69; 1.60]
Shah 2011	0.1310	0.3022	0.9%	1.14 [0.63; 2.06]
Shah 2011	0.6313	0.2787	1.0%	1.88 [1.09; 3.25]
Shah 2011	0.4318	0.1569	1.7%	1.54 [1.13; 2.09]
Shah 2011	0.1310	0.4026	0.6%	1.14 [0.52; 2.52]
Shah 2011	0.3646	0.3271	0.8%	1.44 [0.76; 2.74]
Wang 2015	–0.2485	0.1101	2.2%	0.78 [0.63; 0.97]
**Total (95% CI)**			**100.0%**	**1.00 [0.93; 1.06]**

Heterogeneity: $Tau^2 = 0.0392$; $Chi^2 = 402.16$, df = 53 ($P < 0.01$); $I^2 = 87\%$

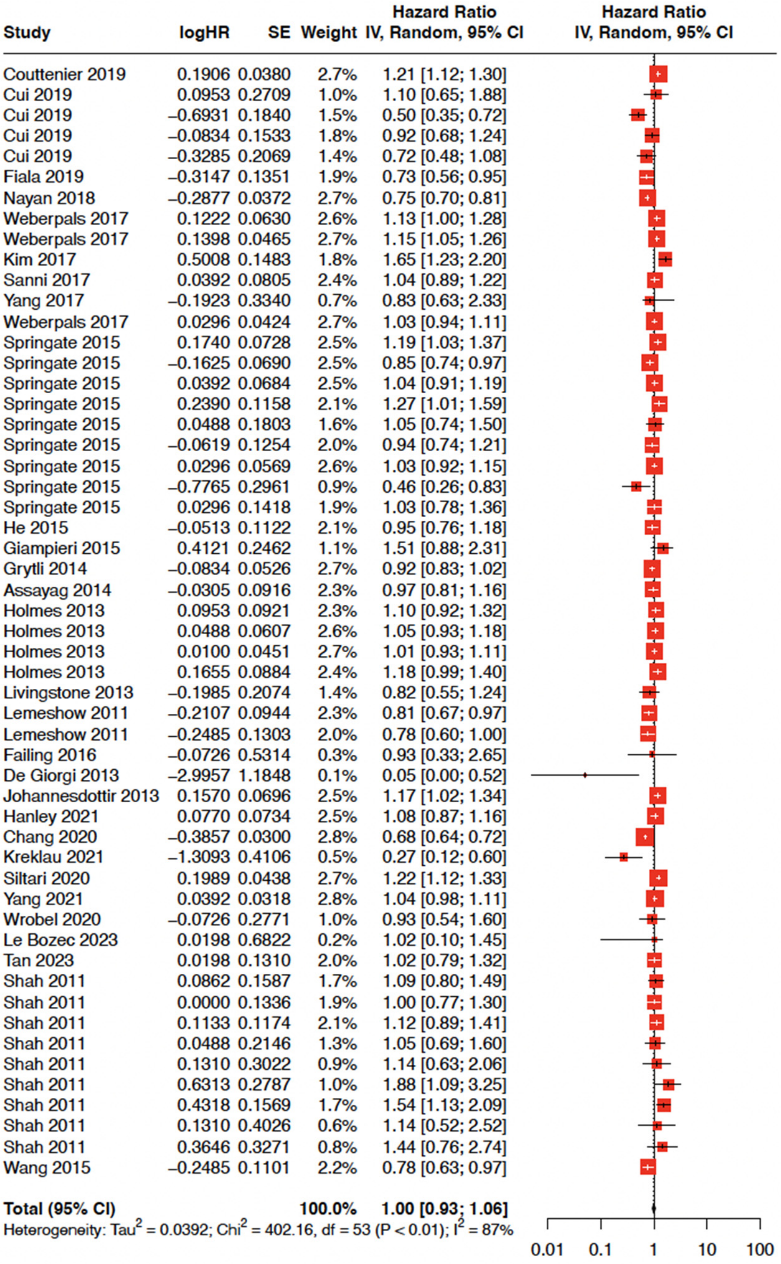

Figure S10. Subgroup Analysis of CSS by Cancer Type

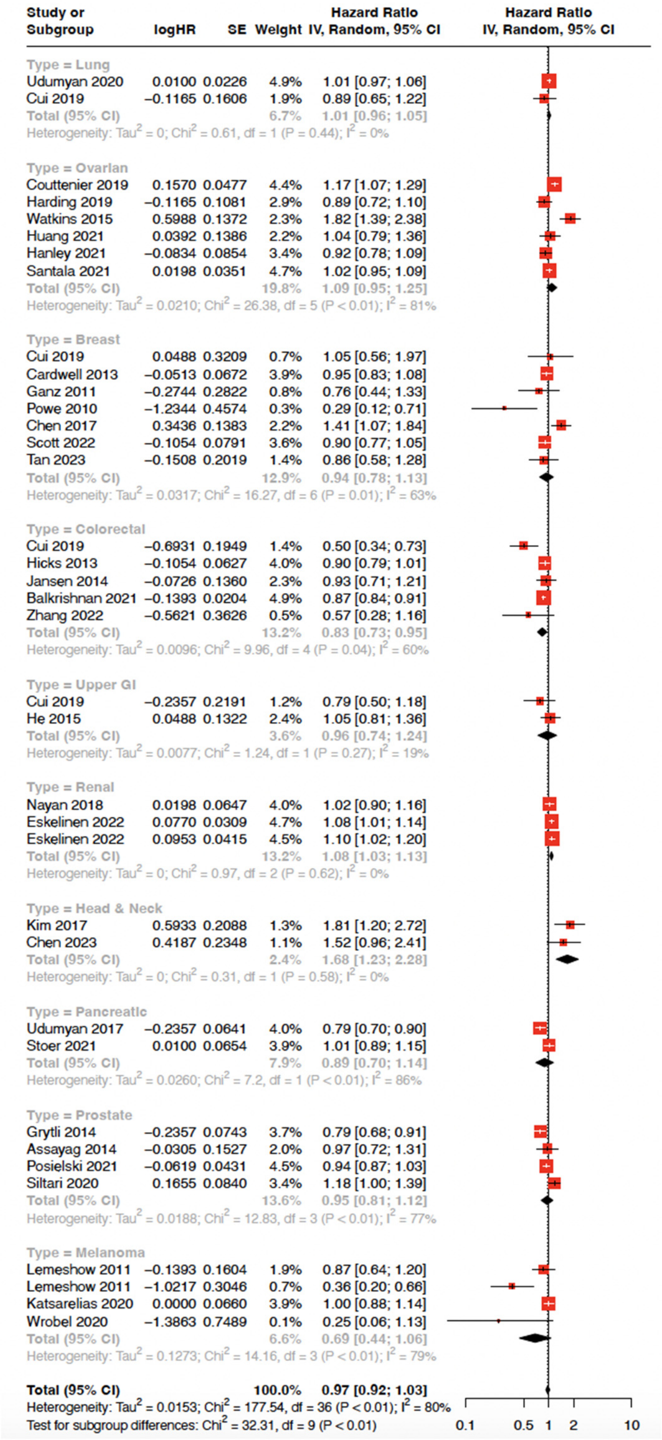

Figure S11. Subgroup Analysis of CSS by Cancer Stage

Study or Subgroup	logHR	SE	Weight	Hazard Ratio IV, Random, 95% CI
**stage_abb = Ear**				
Kim 2017	0.5933	0.2088	5.3%	1.81 [1.20; 2.72]
Assayag 2014	-0.0305	0.1527	7.7%	0.97 [0.72; 1.31]
Ganz 2011	-0.2744	0.2822	3.4%	0.76 [0.44; 1.33]
Powe 2010	-1.2344	0.4574	1.5%	0.29 [0.12; 0.71]
Chen 2017	0.3436	0.1383	8.6%	1.41 [1.07; 1.84]
Katsarelias 2020	0.0000	0.0660	13.7%	1.00 [0.88; 1.14]
Balkrishnan 2021	-0.1393	0.0204	16.3%	0.87 [0.84; 0.91]
**Total (95% CI)**			**56.4%**	**1.02 [0.84; 1.24]**
Heterogeneity: $Tau^2 = 0.0417$; $Chi^2 = 33.56$, df = 6 ($P < 0.01$); $I^2 = 82\%$				
**stage_abb = Adv**				
Grytli 2014	-0.2357	0.0743	13.1%	0.79 [0.68; 0.91]
Posielski 2021	-0.0619	0.0431	15.2%	0.94 [0.87; 1.03]
Eskelinen 2022	0.0953	0.0415	15.3%	1.10 [1.02; 1.20]
**Total (95% CI)**			**43.6%**	**0.94 [0.80; 1.12]**
Heterogeneity: $Tau^2 = 0.0190$; $Chi^2 = 17.08$, df = 2 ($P < 0.01$); $I^2 = 88\%$				
**Total (95% CI)**			**100.0%**	**0.98 [0.88; 1.10]**
Heterogeneity: $Tau^2 = 0.0202$; $Chi^2 = 59.05$, df = 9 ($P < 0.01$); $I^2 = 85\%$				
Test for subgroup differences: $Chi^2 = 0.32$, df = 1 ($P = 0.57$)				

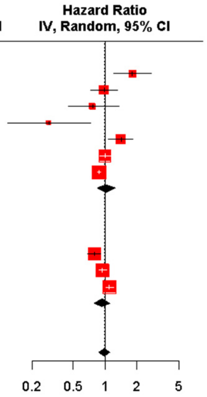

Figure S12. Subgroup Analysis of CSS by Non-selective vs Any Beta Blockers

Study	logHR	SE	Weight	Hazard Ratio IV, Random, 95% CI
Udumyan 2020	0.0770	0.0659	10.4%	1.08 [0.95; 1.23]
Couttenier 2019	0.1740	0.0684	10.3%	1.19 [1.04; 1.36]
Harding 2019	-0.5108	0.1678	7.7%	0.60 [0.43; 0.83]
Udumyan 2017	-0.3011	0.1622	7.9%	0.74 [0.54; 1.02]
Cardwell 2016	0.0100	0.0880	9.9%	1.01 [0.85; 1.20]
Watkins 2015	0.9083	0.3501	3.8%	2.48 [1.25; 4.93]
Assayag 2014	0.0488	0.1923	7.0%	1.05 [0.72; 1.53]
Hicks 2013	-0.0305	0.1675	7.7%	0.97 [0.70; 1.35]
Jansen 2014	0.2700	0.2375	5.9%	1.31 [0.82; 2.08]
Katsarelias 2020	-0.2744	0.2147	6.5%	0.76 [0.50; 1.16]
Udumyan 2022	-0.4155	0.1384	8.5%	0.66 [0.50; 0.86]
Udumyan 2022	0.0583	0.2073	6.7%	1.06 [0.71; 1.60]
Udumyan 2022	-1.0498	0.3391	4.0%	0.35 [0.18; 0.68]
Tan 2023	-0.8675	0.5585	1.9%	0.42 [0.14; 1.25]
Barron 2011	-1.6607	0.5874	1.7%	0.19 [0.06; 0.60]
**Total (95% CI)**			**100.0%**	**0.89 [0.75; 1.05]**
Heterogeneity: $Tau^2 = 0.0648$; $Chi^2 = 59.38$, df = 14 ($P < 0.01$); $I^2 = 76\%$				

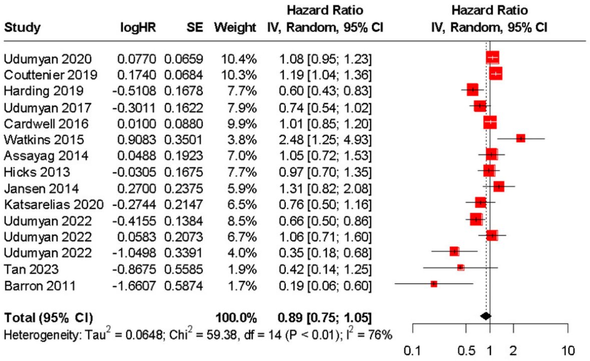

Figure S13. Subgroup Analysis of CSS by Selective vs Any Beta Blockers

Study	logHR	SE	Weight	Hazard Ratio IV, Random, 95% CI
Udumyan 2020	-0.1508	0.0734	8.2%	0.86 [0.75; 1.00]
Udumyan 2020	0.0100	0.0229	11.0%	1.01 [0.96; 1.05]
Couttenier 2019	0.1310	0.0494	9.7%	1.14 [1.03; 1.25]
Harding 2019	0.0296	0.1176	5.6%	1.03 [0.82; 1.30]
Udumyan 2017	-0.1744	0.0672	8.6%	0.84 [0.73; 0.95]
Watkins 2015	0.7178	0.1566	4.1%	2.05 [1.51; 2.79]
Grytli 2014	-0.1985	0.0779	7.9%	0.82 [0.70; 0.95]
Jansen 2014	-0.1625	0.1388	4.7%	0.85 [0.65; 1.12]
Gillis 2021	-0.8916	0.5060	0.6%	0.41 [0.15; 1.09]
Katsarelias 2020	-0.0305	0.0734	8.2%	0.97 [0.84; 1.12]
Udumyan 2022	-0.0943	0.0450	9.9%	0.91 [0.83; 0.99]
Udumyan 2022	-0.0619	0.0789	7.8%	0.94 [0.80; 1.09]
Udumyan 2022	-0.0305	0.1099	6.0%	0.97 [0.78; 1.20]
Tan 2023	-0.0513	0.2091	2.7%	0.95 [0.63; 1.43]
Barron 2011	0.0770	0.1303	5.1%	1.08 [0.84; 1.40]
**Total (95% CI)**			**100.0%**	**0.97 [0.90; 1.05]**
Heterogeneity: $Tau^2 = 0.0135$; $Chi^2 = 54.74$, df = 14 ($P < 0.01$); $I^2 = 74\%$				

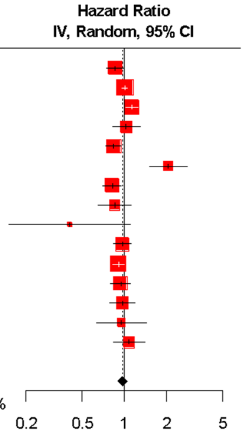

Figure S14. CSS ITB Sensitivity Analysis

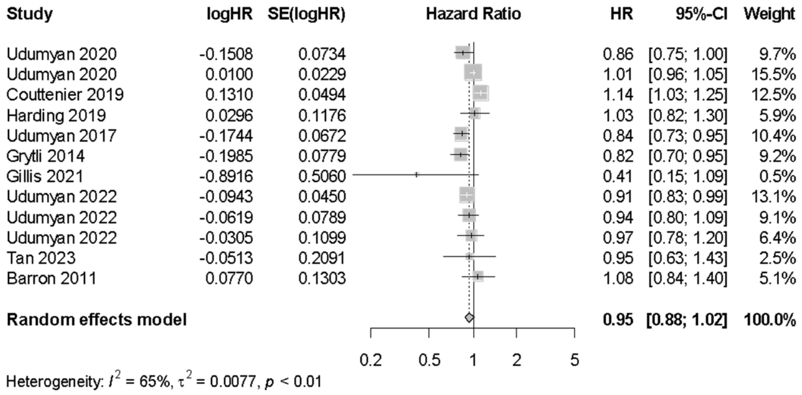

Figure S15. OS Publication Bias

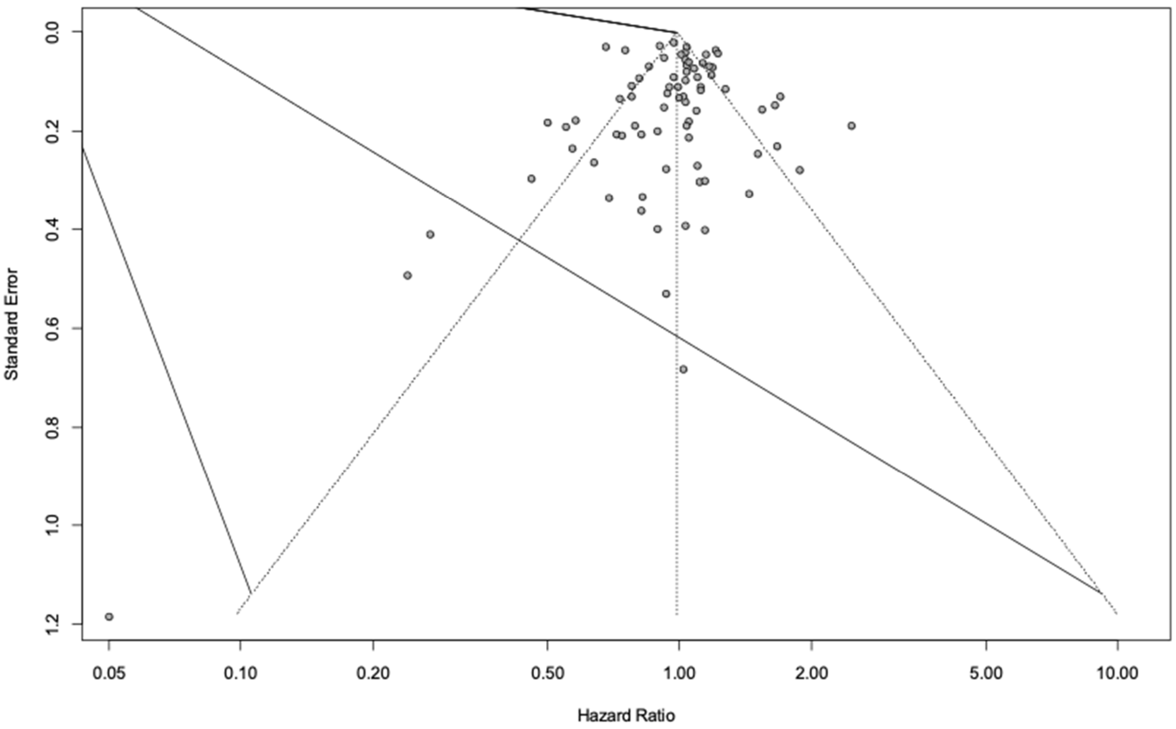

Supplemental Figure S16. CSS Publication Bias

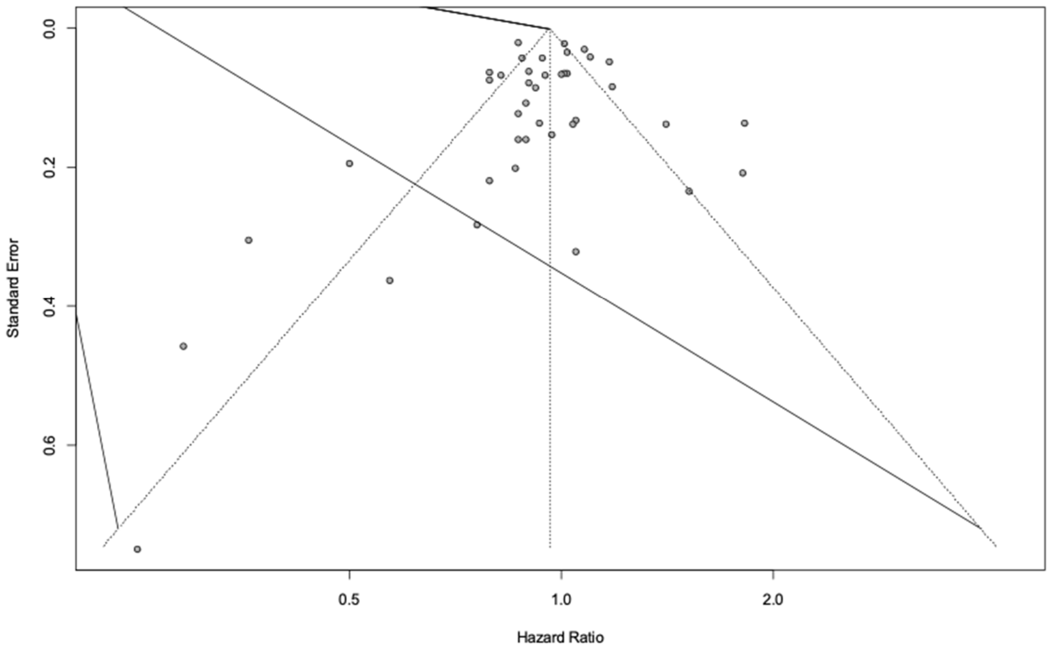

Supplemental Figure S17. PFS Publication Bias

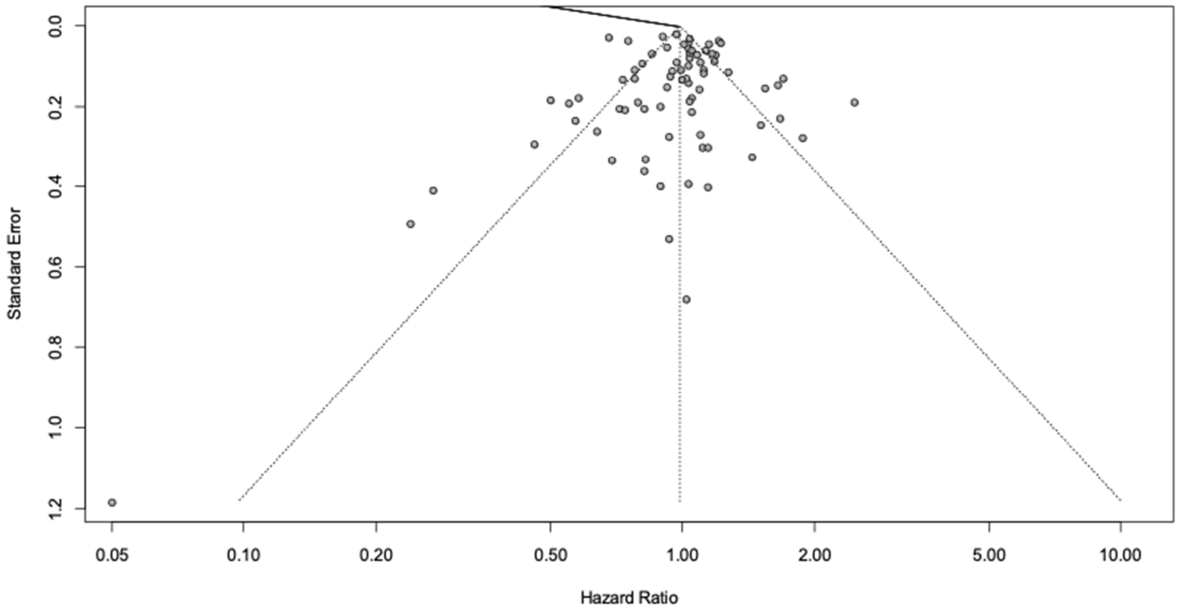

Table S7. GRADE Summary of Findings Table

Summary of findings:

Beta blockers compared to none for adjunct cancer therapy

Patient or population: cancer patients

Intervention: beta blockers

Comparison: none

Outcomes	Anticipated absolute effects* (95% CI)		Relative effect (95% CI)	№ of participants (studies)	Certainty of the evidence (GRADE)	Comments
	**Risk with none**	**Risk with beta blockers**				
Cancer-Specific Survival (CSS)	66 per 1,000	**63 per 1,000** (60 to 66)	**HR 0.95** (0.91 to 1.00)	223376 (37 non-randomised studies)	⊕⊕◯◯ Low[a,b,c,d]	Beta blocker use was not significantly associated with CSS, with meta-analysis yielding a pooled HR of 0.95 (CI 95%: 0.91-1.00).
Progression-Free Survival (PFS)	52 per 1,000	**40 per 1,000** (34 to 48)	**HR 0.78** (0.66 to 0.92)	12322 (27 non-randomised studies)	⊕◯◯◯ Very low[d,e,f,g]	Beta blocker use was associated with improved PFS (HR 0.78 [95% CI: 0.66-0.92]), although I2 of 79.8% suggested a substantial level of heterogeneity in this analysis and Egger's test demonstrated possible publication bias (p=0.03).
Overall Survival (OS)	48 per 1,000	**48 per 1,000** (45 to 50)	**HR 0.99** (0.94 to 1.04)	189965 (56 non-randomised studies)	⊕⊕◯◯ Low[h,i,j]	Beta blocker use was not significantly associated with OS, with a pooled HR of 0.99 (95% CI: 0.94-1.04).

***The risk in the intervention group** (and its 95% confidence interval) is based on the assumed risk in the comparison group and the **relative effect** of the intervention (and its 95% CI).

CI: confidence interval; **HR:** hazard ratio

GRADE Working Group grades of evidence
High certainty: we are very confident that the true effect lies close to that of the estimate of the effect.
Moderate certainty: we are moderately confident in the effect estimate: the true effect is likely to be close to the estimate of the effect, but there is a possibility that it is substantially different.
Low certainty: our confidence in the effect estimate is limited: the true effect may be substantially different from the estimate of the effect.
Very low certainty: we have very little confidence in the effect estimate: the true effect is likely to be substantially different from the estimate of effect.

Explanations

a. 59.4% of studies were at moderate overall risk of bias and 35.1% at serious overall risk of bias using the ROBINS-I scale

b. I^2 77.4% for primary survival analysis

c. 95% CI overlaps 1

d. Egger's test indicates significant funnel plot asymmetry (p = 0.0293) with a bias coefficient of -2.2572, suggesting potential publication bias.

e. 66.7% of studies had serious overall risk of bias on ROBINS-I assessment.

f. I^2 79.8% for primary survival analysis

g. 95% CI overlaps 1

h. 50.0% had moderate overall risk of bias and 42.9% had serious overall risk of bias on ROBINS-I assessment.

i. I^2 84.9% for primary survival analysis

j. 95% CI overlaps 1
